# The predictive value of malnutrition on the prognosis of severe respiratory failure in elderly patients: a multicenter retrospective study based on interpretable machine learning

**DOI:** 10.3389/fnut.2026.1833732

**Published:** 2026-08-07

**Authors:** Xi Wang, Shipeng Ma, Henglu Wang

**Affiliations:** 1 Department of Emergency, Binzhou Medical University Hospital, Binzhou, China; 2 Interventional Vascular Surgery, Binzhou Medical University Hospital, Binzhou, China

**Keywords:** machine learning, nutritional status, predictive model, respiratory failure, risk stratification

## Abstract

**Background:**

Elderly patients (≥65 years) with respiratory failure in the ICU have high mortality. Malnutrition worsens outcomes but is often overlooked. This study investigates nutritional status as a predictor of 28-day mortality and develops an interpretable machine learning-based model for early risk stratification.

**Materials and methods:**

This multicenter retrospective study enrolled elderly patients (≥65 years) with respiratory failure. The internal cohort came from the MIMIC-IV database, and the external validation cohort from Binzhou Medical University Hospital. Multivariable Cox regression analyzed the associations of the Prognostic Nutritional Index (PNI), Hemoglobin-Albumin-Lymphocyte-Platelet (HALP) score, and Geriatric Nutritional Risk Index (GNRI) with 28-day all-cause mortality. Restricted cubic spline and Kaplan–Meier curves explored dose–response relationships and survival differences. The internal cohort was randomly split into training (70%) and testing (30%) sets. The Boruta algorithm combined with LASSO regression selected prognostic features from laboratory tests, vital signs, and demographics. Six machine learning algorithms were built and validated by five-fold cross-validation. Model performance was assessed using calibration curves and decision curve analysis. SHAP was used for interpretability.

**Result:**

Among 1,385 patients, the 28-day mortality was 29.03%. In fully adjusted Cox models, all three indices as continuous variables were significantly inversely associated with mortality risk (PNI: HR = 0.966, 95% CI: 0.952–0.979, *p* < 0.001; HALP: HR = 0.994, 95% CI: 0.990–0.998, *p* = 0.004; GNRI: HR = 0.992, 95% CI: 0.986–0.998, *p* = 0.009). Severe malnutrition (vs. normal) was associated with 33–49% higher risk (all *p* < 0.05). Linear dose–response relationships were confirmed (overall *p* < 0.05, nonlinearity *p* > 0.05). The random forest model achieved the best performance, with AUCs of 0.807 (training), 0.754 (internal test), and 0.734 (external validation). SHAP identified low PNI, elevated BUN, LDH, and RDW as key predictors.

**Conclusion:**

PNI, HALP, and GNRI are independent linear predictors of 28-day mortality in elderly respiratory failure patients. The interpretable machine learning model provides a proof-of-concept tool for risk stratification. However, its low sensitivity limits its current clinical utility, and further optimization is required before clinical implementation.

## Introduction

Respiratory failure accounts for approximately 16% of ICU admissions, with elderly patients (≥65 years) representing 40–50% of cases and carrying persistently high mortality (1–3). The prevalence of nutritional risk in elderly critically ill patients ranges from 40 to 70%, and the coexistence of malnutrition and respiratory failure can increase mortality risk by 2–3 fold. Early identification of high-risk individuals remains a clinical challenge (4).

Nutritional status is a key determinant of prognosis in respiratory diseases (5, 6). The pathophysiological link between malnutrition and adverse outcomes is driven by a vicious cycle of hypercatabolism, inflammation, and muscle wasting. Acute respiratory failure triggers a systemic stress response that increases resting energy expenditure by 20–50% and induces insulin resistance and proteolysis (7, 8). The diaphragm undergoes rapid protein degradation, leading to contractile dysfunction and weaning difficulty (9, 10). Hypoalbuminemia exacerbates pulmonary edema and impairs drug distribution (11), while lymphocytopenia compromises host defense, increasing infection risk (12). Importantly, there is a bidirectional interaction between inflammation and malnutrition: systemic inflammation aggravates nutrient depletion, and worsening nutritional status in turn impairs anti-inflammatory capacity, creating a self-perpetuating cycle (13). Nevertheless, systematic nutritional assessment is often overlooked in clinical practice, and efficient, objective tools capturing nutritional risk and its short-term outcome associations are lacking (14).

Composite nutritional-inflammatory indices derived from routine laboratory tests have gained attention. The Prognostic Nutritional Index (PNI) integrates albumin and lymphocytes (15); the HALP score (hemoglobin, albumin, lymphocytes, platelets) assesses nutrition, inflammation, and hematopoiesis (16); and the Geriatric Nutritional Risk Index (GNRI) is specifically designed for older populations (17). These indices have demonstrated robust prognostic value in various settings, including oncology, severe infections, and surgery (18–21). Other tools such as CONUT (22), NRI (23), mNUTRIC (24), and the NIPS score (25) also show predictive ability, but they often require non-routine tests (e.g., CRP, interleukin-6) or include disease severity components that overlap with clinical judgment. In contrast, PNI, HALP, and GNRI rely solely on routine admission tests (albumin, lymphocytes, hemoglobin, platelets) and basic anthropometrics (height, weight), making them immediately accessible without extra cost or delay. However, their comparative prognostic performance and dose–response relationships with short-term mortality in elderly patients with severe respiratory failure remain unclear.

Therefore, this study not only investigated the independent associations and dose–response relationships of PNI, HALP, and GNRI with 28-day mortality, but also developed and validated a machine learning-based prognostic model incorporating these indices together with other clinical features. Model robustness was assessed using cross-validation and external cohort validation, and SHAP was used to enhance interpretability, aiming to provide clinicians with an objective tool for early nutritional risk identification and timely intervention in elderly patients with severe respiratory failure.

## Materials and methods

### Data source

This study employed a multicenter retrospective cohort design. The internal cohort was derived from the publicly available MIMIC-IV (version 2.2) database, which contains de-identified clinical data from approximately 100,000 intensive care unit (ICU) and emergency department admissions at Beth Israel Deaconess Medical Center in the United States between 2008 and 2019 (26). Its establishment was approved by the Institutional Review Board (IRB: 2001P001699, 0403000206), and, given the fully anonymized nature of the data, the requirement for informed consent was waived. Access to the database was granted to the researchers following completion of the Collaborative Institutional Training Initiative (CITI) program certification. External validation data were obtained from the electronic medical record system of Binzhou Medical University Hospital, encompassing patients admitted with respiratory failure between December 2020 and June 2025 (Supplementary Table S6). The study protocol was reviewed and approved by the hospital’s ethics committee (ethical approval number: 2026-KY-349).

### Study population and inclusion and exclusion criteria

This study focused exclusively on elderly patients (≥65 years of age) with respiratory failure. Cases were identified using International Classification of Diseases, 9th and 10th Revisions (ICD-9/10) codes, as well as the first arterial blood gas analysis performed at admission (PaO₂ < 60 mmHg or PaCO₂ > 50 mmHg with pH < 7.35, with or without mechanical ventilation). Inclusion criteria were: (1) age ≥65 years (elderly patients); (2) first ICU admission; (3) ICU length of stay ≥24 h; (4) complete records of height, weight, lymphocyte count, platelet count, and albumin; (5) exclusion of patients with a history of malignant tumor; (6) exclusion of patients with PaO₂ ≥ 60 mmHg without endotracheal intubation. For the internal (MIMIC-IV) cohort, respiratory failure cases were identified using International Classification of Diseases, 9th and 10th Revisions (ICD-9/10) codes, combined with the first arterial blood gas analysis at admission (PaO₂ < 60 mmHg or PaCO₂ > 50 mmHg with pH < 7.35). For the external validation cohort, respiratory failure was diagnosed based on clinical symptoms (dyspnea, tachypnea, cyanosis), imaging findings (chest X-ray or CT showing bilateral infiltrates or opacities), and arterial blood gas analysis (PaO₂ < 60 mmHg or PaCO₂ > 50 mmHg with pH < 7.35).

### Variable extraction

Data extraction was performed using PostgreSQL (version 13.7.2) and Navicat Premium (version 16) by writing structured query language (SQL) scripts. The extracted variables encompassed six domains: (1) demographic characteristics: age, sex, weight, height, race, (2) comorbidities: acute kidney injury (AKI), chronic kidney disease (CKD), diabetes mellitus (DM), hyperlipidemia (HLD), chronic bronchitis (CB), heart failure (HF), ischemic heart disease (IHD), chronic obstructive pulmonary disease (COPD), and hypertension were identified using ICD-9/10 codes for the internal cohort and by clinical diagnosis from electronic medical records for the external cohort. (3) vital signs: heart rate (HR), non-invasive blood pressure (systolic, diastolic, mean: NBPS, NBPD, NBPM), respiratory rate (RR), oxygen saturation (SpO₂), and temperature in Fahrenheit (Temperaturef). (4) severity of illness scores at admission: Sequential Organ Failure Assessment (SOFA), Acute Physiology Score III (APS III), Simplified Acute Physiology Score II (SAPS II), Oxford Acute Severity of Illness Score (OASIS), Charlson Comorbidity Index, and Acute Physiology and Chronic Health Evaluation II (APACHE II). These were calculated using the standard formulas implemented in the MIMIC-IV database. (5) the first one laboratory blood test results within 24 h of admission:complete blood count (white blood cell count, neutrophil count, lymphocyte count, monocyte count, red blood cell count, hemoglobin, hematocrit, platelet count, red cell distribution width), coagulation profile (international normalized ratio, prothrombin time, activated partial thromboplastin time), biochemistry (albumin, blood urea nitrogen, creatinine, glucose, sodium, potassium, chloride, total carbon dioxide, anion gap, calcium, magnesium, phosphate), liver function tests (alanine aminotransferase, aspartate aminotransferase, total bilirubin, lactate dehydrogenase), and arterial blood gas analysis (pH, partial pressure of oxygen, partial pressure of carbon dioxide, lactate, base excess). (6) treatment modalities and interventions: continuous renal replacement therapy (CRRT), spironolactone (Sa), vasopressors (VP), glucocorticoids (GC), antihypertensive therapy (AHT), antibiotics (ABX), and mechanical ventilation (Ventilation). Sepsis was defined according to the Sepsis-3 criteria: suspected or confirmed infection with an acute increase in SOFA score of ≥2 points. This definition was applied to both cohorts using available clinical and laboratory data. The detailed distribution of these variables is presented in the baseline characteristics table (Table 1). For variables with missing rates below 30% (Supplementary Table S4), multiple imputation was performed using the mice package (version 3.14.0) in R, with 5 imputations and 20 iterations. Predictive mean matching (PMM) was used for continuous variables, and logistic regression for binary variables. A random seed of 123 was set for reproducibility.

**Table 1:** Baseline characteristics of the overall study population and stratified by 28-day ICU survival status in the internal cohort (MIMIC-IV).

Variable	ALL	Survivor	No-survivor	*p*-value
	*N = 1,385*	*N = 983*	*N = 402*	
PNI	35.6 (8.67)	36.7 (8.92)	32.9 (7.40)	<0.001
PNI_group				<0.001
Moderate	222 (16.0%)	175 (17.8%)	47 (11.7%)	
Normal	474 (34.2%)	369 (37.5%)	105 (26.1%)	
Serious	689 (49.7%)	439 (44.7%)	250 (62.2%)	
HALP	25.5 (35.5)	27.4 (37.2)	20.9 (30.4)	0.001
HALP_group				<0.001
Moderate	462 (33.4%)	333 (33.9%)	129 (32.1%)	
Normal	461 (33.3%)	358 (36.4%)	103 (25.6%)	
Serious	462 (33.4%)	292 (29.7%)	170 (42.3%)	
GNRI	98.9 (18.3)	100 (18.0)	96.2 (19.0)	0.001
GNRI_group				0.001
Low	207 (14.9%)	148 (15.1%)	59 (14.7%)	
Moderate	293 (21.2%)	207 (21.1%)	86 (21.4%)	
Normal	668 (48.2%)	497 (50.6%)	171 (42.5%)	
Serious	217 (15.7%)	131 (13.3%)	86 (21.4%)	
Age	75.7 (7.51)	75.4 (7.39)	76.4 (7.76)	0.029
Gender				0.818
F	598 (43.2%)	422 (42.9%)	176 (43.8%)	
M	787 (56.8%)	561 (57.1%)	226 (56.2%)	
Race				0.230
Other races	601 (43.4%)	416 (42.3%)	185 (46.0%)	
WHITE	784 (56.6%)	567 (57.7%)	217 (54.0%)	
BMI(Kg/m^2^)	29.3 (8.10)	29.3 (7.96)	29.1 (8.43)	0.599
Hypertension				0.553
No	907 (65.5%)	649 (66.0%)	258 (64.2%)	
Yes	478 (34.5%)	334 (34.0%)	144 (35.8%)	
AKI				<0.001
No	461 (33.3%)	367 (37.3%)	94 (23.4%)	
Yes	924 (66.7%)	616 (62.7%)	308 (76.6%)	
CKD				0.792
No	942 (68.0%)	666 (67.8%)	276 (68.7%)	
Yes	443 (32.0%)	317 (32.2%)	126 (31.3%)	
DM				0.996
No	856 (61.8%)	607 (61.7%)	249 (61.9%)	
Yes	529 (38.2%)	376 (38.3%)	153 (38.1%)	
HLD				0.040
No	724 (52.3%)	496 (50.5%)	228 (56.7%)	
Yes	661 (47.7%)	487 (49.5%)	174 (43.3%)	
CB				0.802
No	1,069 (77.2%)	761 (77.4%)	308 (76.6%)	
Yes	316 (22.8%)	222 (22.6%)	94 (23.4%)	
HF				0.906
No	739 (53.4%)	526 (53.5%)	213 (53.0%)	
Yes	646 (46.6%)	457 (46.5%)	189 (47.0%)	
IHD				0.636
No	708 (51.1%)	498 (50.7%)	210 (52.2%)	
Yes	677 (48.9%)	485 (49.3%)	192 (47.8%)	
COPD				0.577
No	1,022 (73.8%)	730 (74.3%)	292 (72.6%)	
Yes	363 (26.2%)	253 (25.7%)	110 (27.4%)	
Sofa	7.19 (3.85)	6.67 (3.68)	8.45 (3.98)	<0.001
APSIII	55.7 (22.4)	52.2 (20.7)	64.1 (24.3)	<0.001
SAPSII	47.0 (13.8)	45.1 (13.2)	51.6 (14.4)	<0.001
OASIS	37.7 (8.52)	36.8 (8.21)	40.1 (8.80)	<0.001
Charlson	6.67 (2.45)	6.56 (2.37)	6.93 (2.64)	0.015
APACHEII	22.9 (7.63)	22.1 (7.46)	24.9 (7.71)	<0.001
HR (beats/min)	89.8 (21.5)	89.3 (21.9)	91.1 (20.7)	0.143
NBPS (mmHg)	121 (26.0)	122 (26.2)	117 (25.1)	0.001
NBPD (mmHg)	69.5 (19.8)	70.1 (19.9)	68.2 (19.5)	0.111
NBPM (mmHg)	83.9 (30.3)	84.1 (20.1)	83.6 (46.6)	0.831
RR (insp/min)	96.8 (23.9)	97.2 (28.2)	95.9 (5.15)	0.151
SPO2 (%)	20.7 (6.59)	20.5 (6.48)	21.2 (6.84)	0.059
Temperaturef (°F)	98.2 (3.23)	98.2 (3.71)	98.2 (1.51)	0.970
Lymphocyte_count (%)	1.23 (1.22)	1.32 (1.35)	1.01 (0.78)	<0.001
HCT (%)	32.8 (7.45)	33.1 (7.35)	32.0 (7.63)	0.016
Hb (g/dL)	105 (24.7)	106 (24.6)	102 (24.8)	0.002
PLT (K/μL)	200 (107)	201 (103)	196 (116)	0.436
RDW (%)	15.5 (2.46)	15.1 (2.21)	16.2 (2.84)	<0.001
RBC (m/uL)	3.54 (0.85)	3.58 (0.84)	3.44 (0.88)	0.005
WBC (K/μL)	14.4 (10.3)	14.3 (10.9)	14.5 (8.88)	0.672
Neutrophil_count (%)	11.7 (7.70)	11.3 (7.26)	12.8 (8.61)	0.002
ALB (g/L)	29.5 (6.01)	30.1 (5.81)	27.9 (6.20)	<0.001
AG (mEq/L)	15.2 (5.06)	14.8 (4.71)	16.0 (5.73)	<0.001
Ca (mg/dL)	8.33 (0.82)	8.36 (0.78)	8.25 (0.91)	0.030
CL (mEq/L)	103 (6.86)	103 (6.62)	102 (7.38)	0.014
Glu (mg/dL)	166 (85.5)	165 (87.2)	169 (81.2)	0.451
K (mEq/L)	4.35 (0.80)	4.32 (0.78)	4.44 (0.85)	0.017
Na (mEq/L)	139 (5.90)	139 (5.68)	138 (6.42)	0.636
TCO2 (mEq/L)	24.2 (6.28)	24.4 (5.99)	23.8 (6.92)	0.113
Lac (mmol/L)	2.54 (2.24)	2.37 (1.96)	2.95 (2.77)	<0.001
PCO2 (mmHg)	44.6 (13.2)	44.3 (12.6)	45.4 (14.4)	0.176
PH (pH units)	7.34 (0.11)	7.34 (0.10)	7.32 (0.12)	<0.001
PO2 (mmHg)	115 (102)	120 (106)	101 (90.0)	0.001
INR (ratio)	1.54 (0.84)	1.49 (0.78)	1.66 (0.94)	0.002
PT (seconds)	16.8 (9.31)	16.2 (8.76)	18.1 (10.4)	0.001
APTT (seconds)	40.7 (28.5)	40.2 (28.4)	41.9 (28.8)	0.327
ALT (IU/L)	140 (483)	132 (452)	161 (552)	0.362
AST (IU/L)	213 (975)	190 (649)	272 (1498)	0.290
TB (mg/dL)	1.17 (2.20)	1.02 (1.80)	1.52 (2.92)	0.002
CRE (mg/dL)	1.72 (1.59)	1.62 (1.49)	1.97 (1.78)	<0.001
BUN (mg/dL)	34.8 (26.5)	32.0 (24.6)	41.7 (29.5)	<0.001
LDH (U/L)	548 (1172)	496 (973)	675 (1548)	0.032
BE (mEq/L)	−2.15 (6.03)	−1.85 (5.65)	−2.87 (6.83)	0.008
Mg (mg/dL)	2.07 (0.85)	2.09 (0.97)	2.04 (0.44)	0.174
Phosphate	4.09 (1.63)	3.92 (1.48)	4.52 (1.89)	<0.001
Monocyte_count (%)	0.96 (1.54)	0.96 (1.74)	0.97 (0.84)	0.868
Ventilation				0.001
No	62 (4.48%)	32 (3.26%)	30 (7.46%)	
Yes	1,323 (95.5%)	951 (96.7%)	372 (92.5%)	
CRRT				<0.001
No	1,156 (83.5%)	866 (88.1%)	290 (72.1%)	
Yes	229 (16.5%)	117 (11.9%)	112 (27.9%)	
Sa				<0.001
No	119 (8.59%)	103 (10.5%)	16 (3.98%)	
Yes	1,266 (91.4%)	880 (89.5%)	386 (96.0%)	
VP				<0.001
No	246 (17.8%)	211 (21.5%)	35 (8.71%)	
Yes	1,139 (82.2%)	772 (78.5%)	367 (91.3%)	
GC				0.003
No	820 (59.2%)	607 (61.7%)	213 (53.0%)	
Yes	565 (40.8%)	376 (38.3%)	189 (47.0%)	
AHT				0.025
No	166 (12.0%)	105 (10.7%)	61 (15.2%)	
Yes	1,219 (88.0%)	878 (89.3%)	341 (84.8%)	
ABX				0.222
No	65 (4.69%)	51 (5.19%)	14 (3.48%)	
Yes	1,320 (95.3%)	932 (94.8%)	388 (96.5%)	
Survival_time	22.4 (9.28)	28.0 (0.00)	8.78 (5.88)	<0.001

Data are presented as mean (standard deviation) for continuous variables and number (percentage) for categorical variables. Between-group comparisons were performed using the Chi-square test for categorical variables and Student’s t-test or Mann–Whitney U test for continuous variables, as appropriate. All enrolled patients were aged 65 years or older, with a mean age of 75.7 years. PNI, Prognostic Nutritional Index; HALP, Hemoglobin, Albumin, Lymphocyte, and Platelet score; GNRI, Geriatric Nutritional Risk Index; BMI, Body Mass Index; AKI, Acute Kidney Injury; CKD, Chronic Kidney Disease; DM, Diabetes Mellitus; HLD, Hyperlipidemia; CB, Chronic bronchitis; HF, Heart Failure; IHD, Ischemic Heart Disease; COPD, Chronic Obstructive Pulmonary Disease; SOFA, Sequential Organ Failure Assessment; APS III, Acute Physiology Score III; SAPS II, Simplified Acute Physiology Score II; OASIS, Oxford Acute Severity of Illness Score; Charlson, Charlson Comorbidity Index; APACHE II, Acute Physiology and Chronic Health Evaluation II; HR, Heart Rate; NBPS/NBPD/NBPM, Non-Invasive Blood Pressure Systolic/Diastolic/Mean; RR, Respiratory Rate; SpO₂, Oxygen Saturation; Temperaturef, Temperature in Fahrenheit; Lymphocyte_count, Lymphocyte count; HCT, Hematocrit; Hb, Hemoglobin; PLT, Platelet count; RDW, Red Cell Distribution Width; RBC, Red Blood Cell count; WBC, White Blood Cell count; Neutrophil_count, Neutrophil count; ALB, Albumin; AG, Anion Gap; Ca, Total Calcium; CL, Chloride; Glu, Glucose; K, Potassium; Na, Sodium; TCO₂, Total Carbon Dioxide; Lac, Lactate; PCO₂, Partial Pressure of Carbon Dioxide; PH, Potential of Hydrogen; PO₂, Partial Pressure of Oxygen; INR, International Normalized Ratio; PT, Prothrombin Time; APTT, Activated Partial Thromboplastin Time; ALT, Alanine Aminotransferase; AST, Aspartate Aminotransferase; TB, Total Bilirubin; CRE, Creatinine; BUN, Blood Urea Nitrogen; LDH, Lactate Dehydrogenase; BE, Base Excess; Mg, Magnesium; Phosphate, Phosphate; Monocyte_count, Monocyte count; CRRT, Continuous Renal Replacement Therapy; Sa, Spironolactone; VP, Vasopressor; GC, Glucocorticoid; AHT, Antihypertensive Therapy; ABX, Antibiotics.

### Definition of research variables and endpoints

Nutritional status was assessed using three composite indices. The Prognostic Nutritional Index (PNI) was calculated as serum albumin (g/L) + 5 × total lymphocyte count (×10^9^/L), and was categorized as normal (>38), moderate malnutrition (35–38), or severe malnutrition (<35) (15). The HALP score was computed as hemoglobin (g/L) × albumin (g/L) × lymphocytes (×10^9^/L) / platelets (×10^9^/L), and was stratified by tertiles into normal (>23.60), moderate (10.56–23.60), and severe (<10.56) malnutrition categories (16). The Geriatric Nutritional Risk Index (GNRI) was defined as 1.489 × albumin (g/L) + 41.7 × (actual body weight / ideal body weight). Ideal body weight was calculated using the Lorentz formula: for males, height (cm) - 100 − [(height (cm) − 150) / 4]; for females, height (cm) - 100 − [(height (cm) − 150) / 2.5] (27). GNRI was graded as follows: ≥98 (no risk), 92–98 (low risk), 82–92 (moderate risk), and <82 (high risk). The study endpoint was defined as 28-day ICU mortality.

### Descriptive statistics and group comparisons

Continuous variables were presented as mean ± standard deviation. For between-group comparisons, the t-test or analysis of variance (ANOVA) was applied when data met the assumptions of normality and homogeneity of variance; otherwise, the Mann–Whitney U test or Kruskal-Wallis test was used. Categorical variables were described using frequencies and percentages, and intergroup comparisons were performed using the Pearson chi-square test or Fisher’s exact test, as appropriate.

### Multivariable Cox regression and survival analysis

Multivariable Cox proportional hazards models were used to evaluate the associations between the three nutritional indices (PNI, HALP score, GNRI) and 28-day all-cause mortality. Three sequentially adjusted models were constructed: Model 1 was unadjusted; Model 2 was adjusted for demographic characteristics (age, sex, race, BMI), baseline comorbidities, and treatments associated with the study endpoint; Model 3 was further adjusted for variables that differed significantly between survivors and non-survivors at baseline, including laboratory findings, vital signs, admission severity scores, and therapeutic interventions. To mitigate multicollinearity, variance inflation factors (VIF) were calculated separately for each cohort for all variables in Model 3; variables with VIF > 5 were excluded on a per-cohort basis (Supplementary Tables S3). The three nutritional indices were analyzed in separate Cox models (each index entered individually), not simultaneously, to avoid multicollinearity. The proportional hazards assumption was tested using Schoenfeld residuals for all covariates and the global model. The test yielded *p*-values > 0.05 for the global model and for each of the three nutritional markers (PNI, HALP, GNRI), confirming that the assumption was satisfied (see Supplementary Table S5; Supplementary Figure S3). Restricted cubic spline (RCS) analyses with four knots (5th, 35th, 65th, and 95th percentiles of each index distribution) were performed to flexibly model dose–response relationships. Kaplan–Meier survival curves were generated, and between-group differences were compared using the Log-rank test.

### Feature selection and machine learning model development

The internal MIMIC-IV cohort was randomly divided into a training set (70%) and an internal testing set (30%) using the “rsample” package in R with a random seed of 1. In the training set, the Boruta algorithm combined with least absolute shrinkage and selection operator (LASSO) regression was employed to select prognostic features from laboratory tests, vital signs, and demographic characteristics, LASSO regression is designed to handle collinearity by shrinking coefficients of redundant predictors. Features identified by both methods were retained for subsequent model development. Based on these core features, six machine learning algorithms were used to construct prediction models: Decision Tree (DT), Random Forest (RF), Logistic Regression (LR), Naive Bayes (NB), Multilayer Perceptron (MLP), and K-Nearest Neighbors (KNN). Five-fold cross-validation was applied to the training set to assess model stability.

### Model performance evaluation

Model comparisons were performed using the DeLong test. Discriminative ability was evaluated using area under the receiver operating characteristic curve (AUC), sensitivity, specificity, accuracy, and F1 score. Calibration was assessed using calibration curves, and clinical utility was evaluated using decision curve analysis (DCA). Optimal probability thresholds were determined using precision-recall (PR) curves.

### Model interpretability

To elucidate the contribution of individual features to the predictions of the optimal model, the SHapley Additive exPlanations (SHAP) method was employed. This game-theory-based approach attributes the change in a model’s prediction to each input feature, providing a consistent and interpretable measure of feature importance, thereby enhancing the transparency and credibility of the model’s decision-making process (28).

### Software and significance level

All statistical analyses were performed using R software (version 4.2.2) and DecisionLinnc (version 1.0). A two-sided *p* < 0.05 was considered statistically significant.

## Result

### Baseline characteristics of study participants

A total of 1,385 patients with respiratory failure were included, with a 28-day all-cause mortality rate of 29.03%. The cohort had a mean age of 75.7 ± 7.51 years (consistent with an elderly population), and 56.8% were male. As shown in Table 1, non-survivors had significantly lower mean values of PNI (32.9 ± 7.40 vs. 36.7 ± 8.92), HALP score (20.9 ± 30.4 vs. 27.4 ± 37.2), and GNRI (96.2 ± 19.0 vs. 100 ± 18.0) compared with survivors (all *p* < 0.01). The proportion of patients with severe malnutrition was substantially higher among non-survivors than survivors across all three indices (PNI: 62.2% vs. 44.7%; HALP: 42.3% vs. 29.7%; GNRI: 21.4% vs. 13.3%; all *p* < 0.001). Non-survivors also had higher illness severity scores, including SOFA, APS III, SAPS II, OASIS, and APACHE II (all p < 0.001), and worse laboratory parameters, such as lower albumin and hemoglobin, and higher RDW, neutrophil count, lactate, BUN, creatinine, and phosphorus (all *p* < 0.05). Regarding therapeutic interventions, non-survivors were significantly more likely to receive continuous renal replacement therapy (CRRT) (27.9% vs. 11.9%) and vasopressors (91.3% vs. 78.5%) (both *p* < 0.001).

### The relationship between nutritional status and 28 day ICU mortality in patients with respiratory failure

Multivariable Cox regression analysis showed that all three nutritional indices—PNI, HALP score, and GNRI—were independent predictors of 28-day all-cause mortality in patients with respiratory failure, exhibiting clear dose–response relationships (Table 2). In the fully adjusted model (Model 3), all three indices as continuous variables were significantly inversely associated with mortality risk, i.e., higher index values (better nutritional status) correlated with lower mortality risk. Each one-unit increase in PNI was associated with a 3.4% reduction in mortality risk (HR = 0.966, 95% CI: 0.952–0.979, *p* < 0.001); each one-unit increase in HALP score was associated with a 0.6% reduction (HR = 0.994, 95% CI: 0.990–0.998, *p* = 0.004); and each one-unit increase in GNRI was associated with a 0.8% reduction (HR = 0.992, 95% CI: 0.986–0.998, *p* = 0.009). These associations remained consistent in the unadjusted model (Model 1) and the model adjusted only for demographic characteristics (Model 2). When converting continuous indices into categorical variables for risk stratification, a clear clinical risk gradient was revealed. Using normal nutritional status as the reference, the severe malnutrition group was consistently associated with the highest mortality risk. In the fully adjusted model, patients with severe malnutrition according to PNI had a 1.329-fold higher risk of mortality compared to the normal group (HR = 1.329, 95% CI: 1.037–1.703, *p* = 0.025); those with severe malnutrition by HALP score had a 1.487-fold higher risk (HR = 1.487, 95% CI: 1.142–1.936, *p* = 0.003); and those with severe malnutrition by GNRI had a 1.493-fold higher risk (HR = 1.493, 95% CI: 1.120–1.989, *p* = 0.006).

**Table 2:** Analysis of the association between 3 nutritional status indicators and 28 day all-cause mortality rate in respiratory failure patients through multiple COX regression analysis.

Characteristic	Model 1		Model 2		Model 3	
	HR (95% CI)	*p*-value	HR (95% CI)	*p*-value	HR (95% CI)	*p*-value
Continuous_PNI	0.949 (0.936, 0.962)	<0.001	0.959 (0.946, 0.972)	<0.001	0.966 (0.952, 0.979)	<0.001
PNI_group						
Normal	Reference		Reference		Reference	
Moderate	0.957 (0.678, 1.350)	0.802	0.896 (0.634, 1.268)	0.536	0.865 (0.610, 1.227)	0.416
Severe	1.812 (1.442, 2.278)	<0.001	1.554 (1.221, 1.953)	<0.001	1.329 (1.037, 1.703)	0.025
Continuous_HALP	0.993 (0.989, 0.999)	0.002	0.993 (0.989, 0.997)	0.001	0.994 (0.990, 0.998)	0.004
HALP_group						
Normal	Reference		Reference		Reference	
Moderate	1.279 (0.987, 1.659)	0.063	1.414 (1.089, 1.837)	0.009	1.300 (0.991, 1.705)	0.059
Severe	1.800 (1.408, 2.302)	0.001	1.740 (1.357, 2.231)	<0.001	1.487 (1.142, 1.936)	0.003
Continuous_GNRI	0.989 (0.984, 0.995)	<0.001	0.976 (0.965, 0.987)	<0.001	0.992 (0.986, 0.998)	0.009
GNRI_group						
Normal	Reference		Reference		Reference	
Low	1.098 (0.815, 1.480)	0.537	1.250 (0.901, 1.733)	0.031	1.258 (0.923, 1.715)	0.146
Moderate	1.178 (0.909, 1.526)	0.216	1.250 (0.923, 1.715)	0.925	1.070 (0.815, 1.406)	0.626
Severe	1.713 (1.332, 2.221)	<0.001	1.842 (1.302, 2.606)	0.001	1.493 (1.120, 1.989)	0.006

PNI, Prognostic Nutritional Index (normal >38, moderate 35–38, severe <35); HALP, Hemoglobin, Albumin, Lymphocyte, Platelet score (normal >23.60, moderate 10.56–23.60, severe <10.56); GNRI, Geriatric Nutritional Risk Index (no risk ≥98, low risk 92–98, moderate risk 82–92, severe risk <82). The reference group for each index is the normal (or no risk) category, with HR set as Reference. Model 1: unadjusted. Model 2: adjusted for demographic characteristics (gender, age, race, and BMI; for GNRI analysis, BMI was excluded as it is a component of the index) and treatment interventions (ventilation, GC, Sa, VP, AHT, AKI, CRRT). Model 3: further adjusted based on Model 2 for ventilation, GC, HLD, NBPS, APTT, Sa, SpO₂, VP, AHT, PO₂, AKI, calcium, LDH, total bilirubin, Charlson comorbidity index, age, potassium, RDW, CRRT, chloride, BUN, OASIS, phosphate, lactate, creatinine, SOFA, anion gap, APACHE II, SAPS II, and APS III.

### Linear relationship between nutritional status and 28 day ICU mortality in patients with respiratory failure

**Figure 1:**
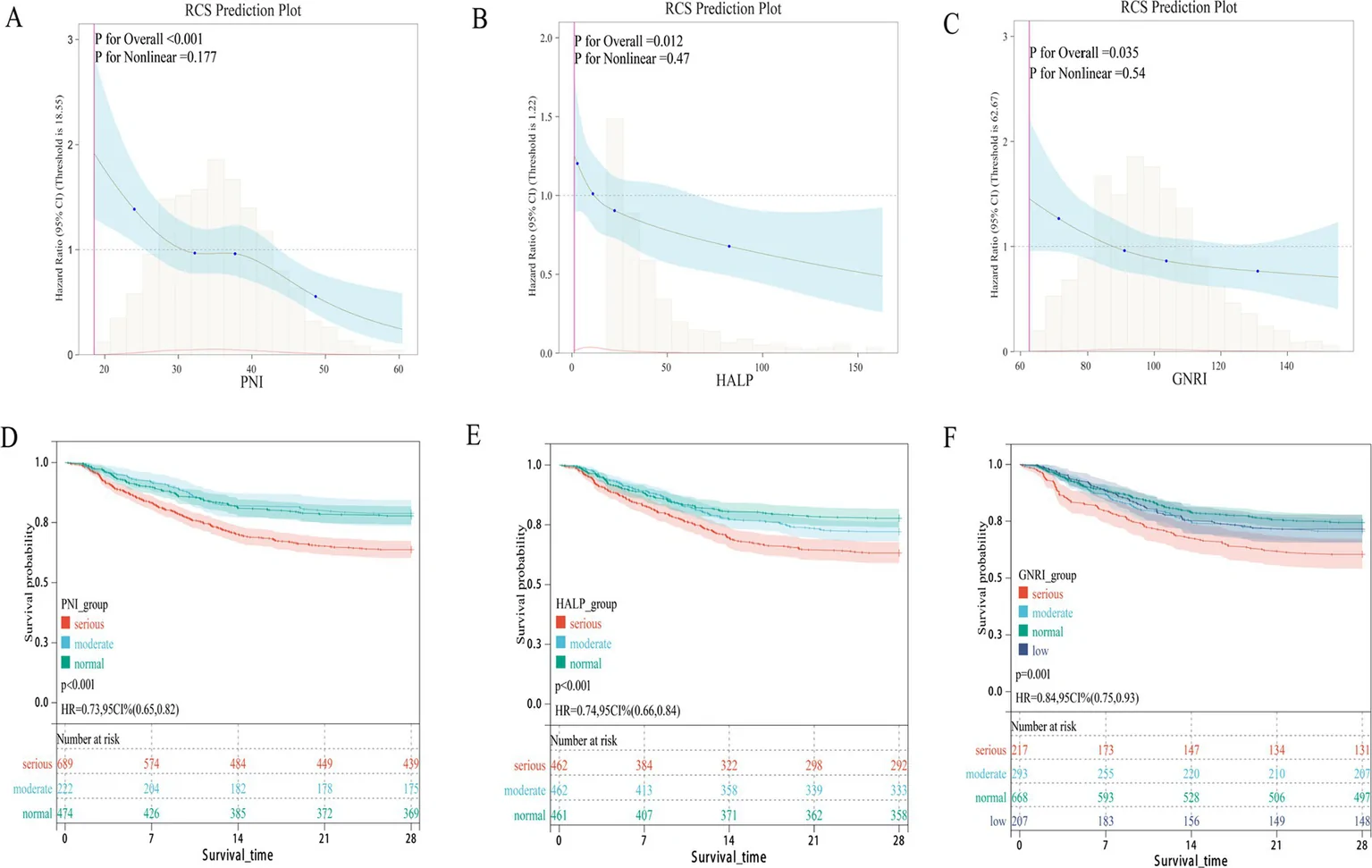
Association of nutritional indices with 28-day mortality risk in elderly respiratory failure patients from the internal (MIMIC-IV) cohort. **(A–C)** Restricted cubic spline (RCS) analysis demonstrates the continuous, dose–response relationship between the three nutritional indices as continuous variables and the adjusted hazard ratio (*HR*) for 28-day all-cause mortality in the internal cohort. The solid line represents the estimated *HR*, with the shaded area indicating the 95% confidence interval. *P* for overall indicates the significance of the entire curve, while *P* for nonlinear tests for deviation from linearity. **(D–F)** Kaplan–Meier survival curves stratified by nutritional status (normal, moderate malnutrition, and severe malnutrition) according to each index in the internal cohort. The Log-rank test *p*-values are displayed, demonstrating significant survival differences across the nutritional strata. PNI, Prognostic Nutritional Index; HALP, Hemoglobin, Albumin, Lymphocyte, Platelet score; GNRI, Geriatric Nutritional Risk Index.

RCS regression analyses further characterized the shape of the associations between the continuous nutritional indices and mortality risk (Figures 1A–C). These analyses demonstrated a significant overall association for PNI, HALP score, and GNRI with 28-day mortality risk (P for overall < 0.05 for all), and all associations were linear in nature (P for nonlinearity > 0.05 for all), indicating a continuous increase in mortality risk without a significant threshold effect. This indicates that mortality risk increases continuously with declining nutritional status, without evidence of a significant threshold effect. Kaplan–Meier survival curves illustrated survival disparities among patients stratified by nutritional status (Figures 1D–F). Log-rank tests confirmed significant differences in survival curves across categories defined by PNI, HALP score, and GNRI (*p* < 0.001 for all). A gradient of decreasing 28-day survival probability was observed with increasing severity of malnutrition, further corroborating the significant impact of nutritional status on short-term prognosis in patients with respiratory failure.

### External cohort verification

**Figure 2:**
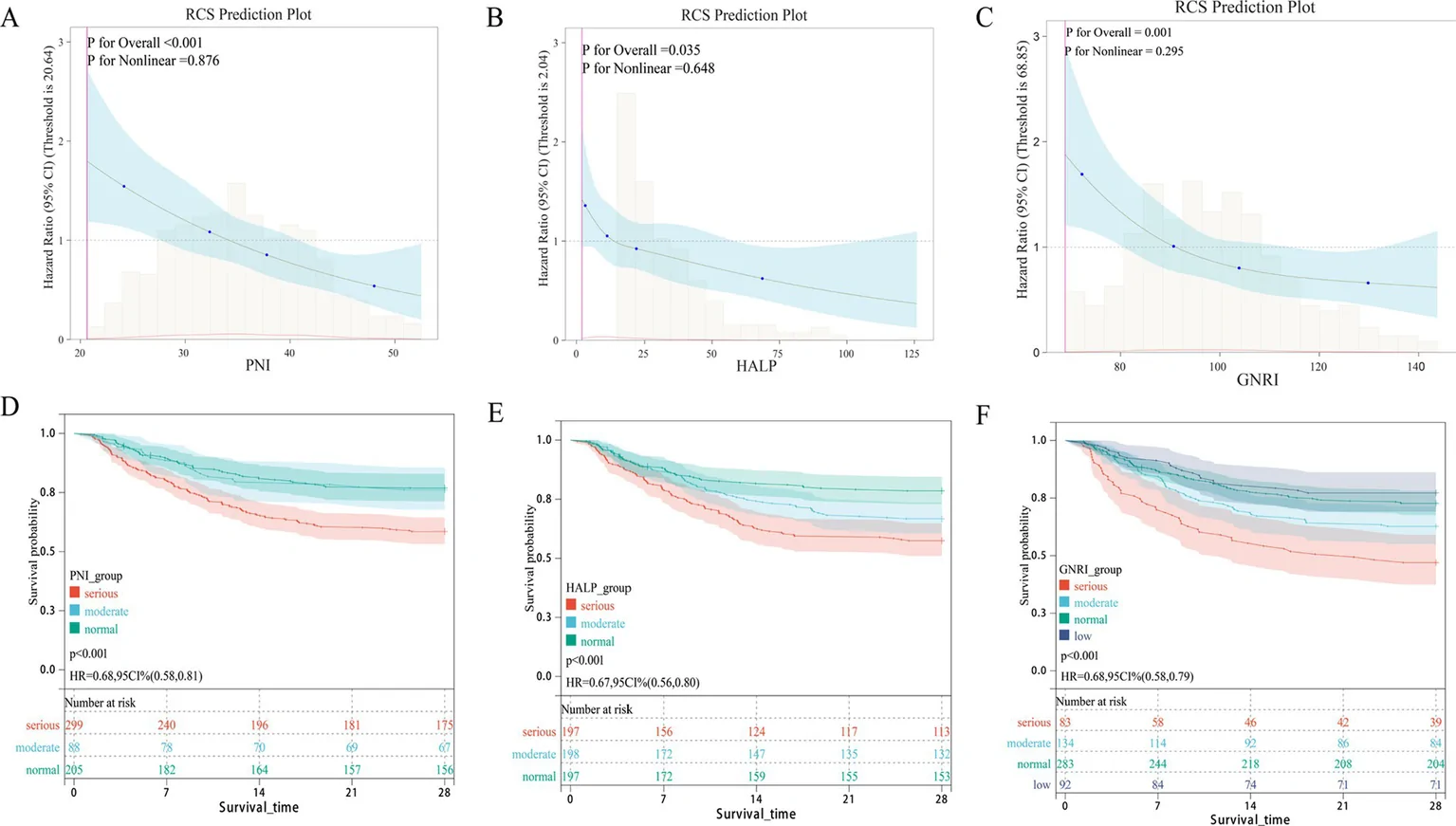
Association of nutritional indices with 28-day mortality risk in elderly respiratory failure patients from the external validation cohort. **(A–C)** Restricted cubic spline (RCS) analysis demonstrates the continuous, dose–response relationship between the three nutritional indices as continuous variables and the adjusted hazard ratio (*HR*) for 28-day all-cause mortality in the internal cohort. The solid line represents the estimated *HR*, with the shaded area indicating the 95% confidence interval. *P* for overall indicates the significance of the entire curve, while *P* for nonlinear tests for deviation from linearity. **(D–F)** Kaplan–Meier survival curves stratified by nutritional status (normal, moderate malnutrition, and severe malnutrition) according to each index in the internal cohort. The Log-rank test *p*-values are displayed, demonstrating significant survival differences across the nutritional strata. PNI, Prognostic Nutritional Index; HALP, Hemoglobin, Albumin, Lymphocyte, Platelet score; GNRI, Geriatric Nutritional Risk Index.

To assess robustness, an external validation cohort of patients with respiratory failure from Binzhou Medical University Hospital was included (28-day mortality: 32.43%; Supplementary Table S1). Multivariable Cox regression (Table 3) confirmed that the associations between the three nutritional indices and mortality risk were highly consistent with the internal cohort. In the fully adjusted model (Model 3), all three indices as continuous variables were significantly inversely associated with mortality risk: each one-unit increase in PNI, HALP, and GNRI was associated with a 4.3% (HR = 0.957, 95% CI: 0.938–0.976, *p* < 0.001), 1.1% (HR = 0.989, 95% CI: 0.981–0.997, *p* = 0.008), and 1.7% (HR = 0.983, 95% CI: 0.974–0.993, *p* < 0.001) reduction in mortality risk, respectively. When analysed categorically (normal status as reference), severe malnutrition was again associated with the highest risk: PNI (HR = 1.651, 95% CI: 1.159–2.350, *p* = 0.005), HALP (HR = 1.819, 95% CI: 1.218–2.716, *p* = 0.003), and GNRI (HR = 2.159, 95% CI: 1.436–3.245, *p* < 0.001). RCS analyses confirmed linear dose–response relationships (P for overall < 0.05, P for nonlinearity > 0.05 for all; Figures 2A–C), and Kaplan–Meier curves showed significant survival differences across nutritional status groups (log-rank *p* < 0.001 for all; Figures 2D–F). These findings further validate the prognostic value of nutritional status for short-term outcomes in patients with respiratory failure.

**Table 3:** Multiple Cox regression analysis was conducted in the external validation cohort to analyze the association between three nutritional status indicators and 28 day all-cause mortality rate in patients with respiratory failure.

Characteristic	Model 1		Model 2		Model 3	
	HR (95% CI)	*p*-value	HR (95% CI)	*p*-value	HR (95% CI)	*p*-value
Continuous_PNI	0.939 (0.921, 0.958)	<0.001	0.948 (0.930, 0.967)	<0.001	0.957 (0.938, 0.976)	<0.001
PNI_group						
Normal	Reference		Reference		Reference	
Moderate	1.041 (0.623, 1.742)	0.811	1.048 (0.623, 1.763)	0.859	1.015 (0.601, 1.716)	0.955
Severe	2.040 (1.458, 2.854)	<0.001	1.854 (1.309, 2.602)	<0.001	1.651 (1.159, 2.350)	0.005
Continuous_HALP	0.986 (0.977, 0.994)	0.001	0.980 (0.967, 0.992)	<0.001	0.989 (0.981, 0.997)	0.008
HALP_group						
Normal	Reference		Reference		Reference	
Moderate	1.613 (1.095, 2.375)	0.015	1.732 (1.175, 2.554)	0.006	1.596 (1.071, 2.378)	0.022
Severe	2.260 (1.560, 3.274)	<0.001	2.330 (1.601, 3.389)	<0.001	1.819 (1.218, 2.716)	0.003
Continuous_GNRI	0.982 (0.973, 0.991)	<0.001	0.968 (0.953, 0.984)	<0.001	0.983 (0.974, 0.993)	<0.001
GNRI_group						
Normal	Reference		Reference		Reference	
Low	0.809 (0.500, 1.311)	0.390	0.947 (0.561, 1.600)	0.839	0.940 (0.569, 1.552)	0.808
Moderate	1.450 (1.016, 2.071)	0.041	1.664 (1.076, 2.574)	0.022	1.520 (1.037, 2.228)	0.032
Severe	2.402 (1.658, 3.480)	<0.001	2.630 (1.580, 4.377)	<0.001	2.159 (1.436, 3.245)	<0.001

PNI, Prognostic Nutritional Index (normal >38, moderate 35–38, severe <35); HALP, Hemoglobin, Albumin, Lymphocyte, Platelet score (normal >23.41, moderate 10.66–23.41, severe <10.66); GNRI, Geriatric Nutritional Risk Index (no risk ≥98, low risk 92–98, moderate risk 82–92, severe risk <82). The reference group for each index is the normal (or no risk) category, with HR set as Reference. Model 1: unadjusted. Model 2: adjusted for demographic characteristics (gender, age, race, and BMI; for GNRI analysis, BMI was excluded as it is a component of the index) and treatment interventions (ventilation, Sa, VP, AKI, CRRT). Model 3: further adjusted based on Model 2 for temperature, RBC, ventilation, APTT, NBPS, LDH, VP, RDW, Sa, AKI, age, total bilirubin, CRRT, lactate, phosphate, OASIS, SOFA, BUN, APACHE II, creatinine, SAPS II, and APS III.

### Selection of characteristic variables and establishment of models

**Figure 3:**
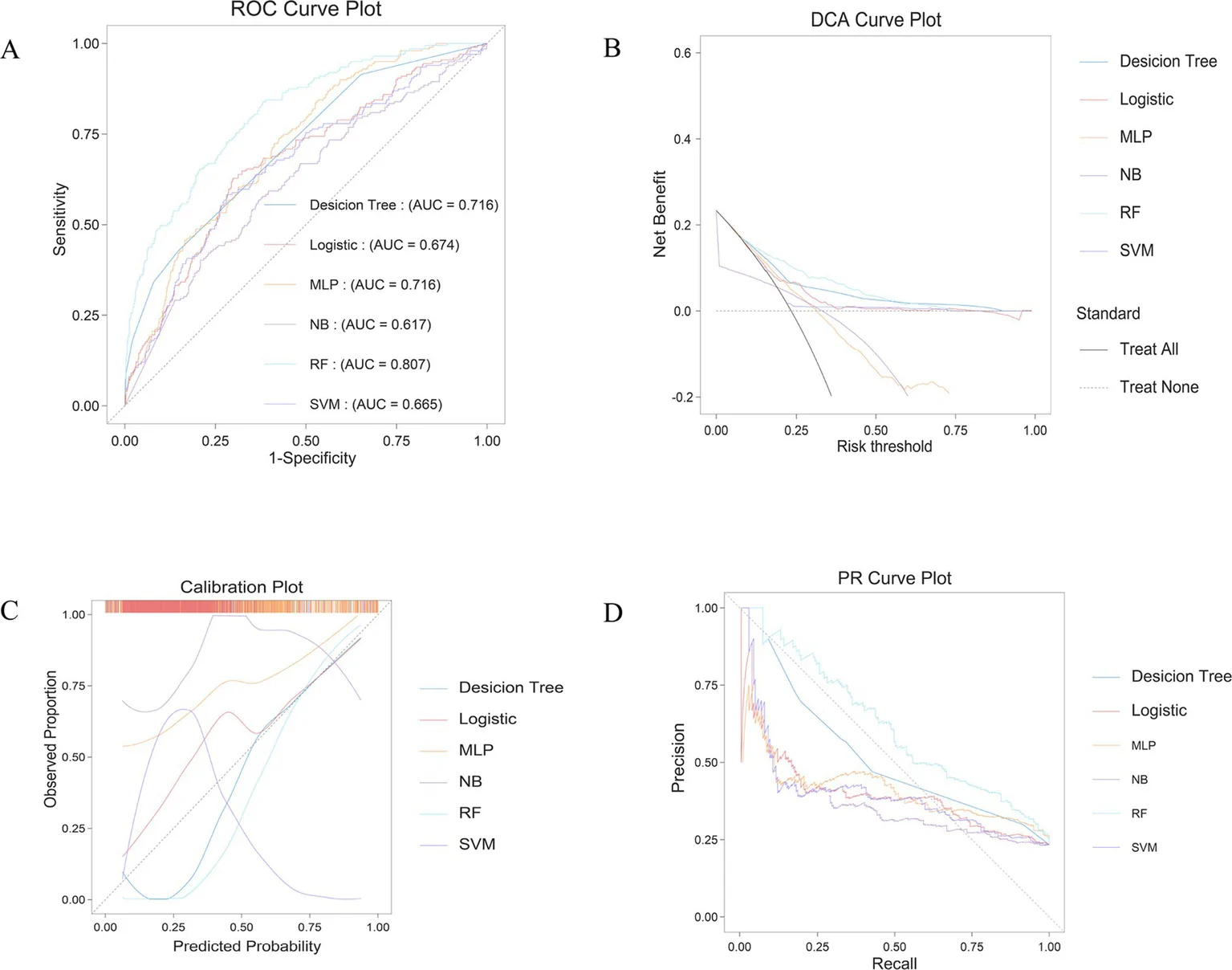
Performance evaluation of six machine learning prediction models on the training set. **(A)** Receiver operating characteristic (ROC) curves. The area under the curve (AUC) with 95% confidence intervals is shown for each model. **(B)** Decision curve analysis (DCA). The net benefit across a range of threshold probabilities is plotted. The gray solid line represents the strategy of “treating all” patients, and the gray dashed line represents “treating none”. A model curve above these lines indicates clinical utility within that probability range. **(C)** Calibration curves. The plots compare predicted probabilities against observed event frequencies. The dotted diagonal line represents perfect calibration. **(D)** Precision-recall (PR) curves. Models: Random Forest (RF), K-Nearest Neighbors (KNN), Logistic Regression (Logistic), Multi-layer Perceptron (MLP), Decision Tree (DT), and Naïve Bayes (NB).

**Figure 4:**
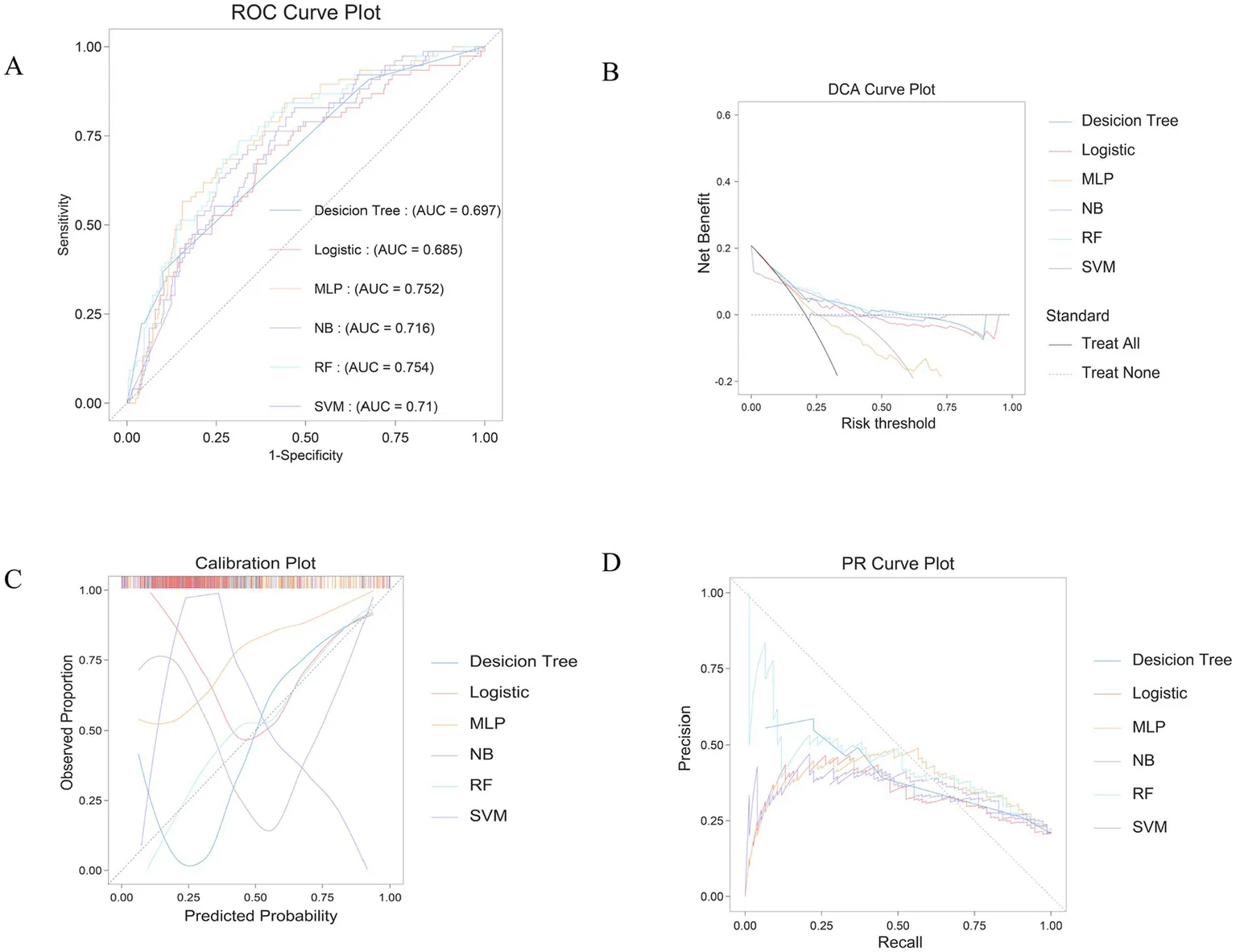
Performance evaluation of six machine learning prediction models on the testing set. **(A)** Receiver operating characteristic (ROC) curves. The area under the curve (AUC) with 95% confidence intervals is shown for each model. **(B)** Decision curve analysis (DCA). The net benefit across a range of threshold probabilities is plotted. The gray solid line represents the strategy of “treating all” patients, and the gray dashed line represents “treating none”. A model curve above these lines indicates clinical utility within that probability range. **(C)** Calibration curves. The plots compare predicted probabilities against observed event frequencies. The dotted diagonal line represents perfect calibration. **(D)** Precision-recall (PR) curves. Models: Random Forest (RF), K-Nearest Neighbors (KNN), Logistic Regression (Logistic), Multi-layer Perceptron (MLP), Decision Tree (DT), and Naïve Bayes (NB).

To develop a parsimonious risk model for prognosis in respiratory failure, all patients in the internal cohort were randomly allocated to a training set and a testing set in a 7:3 ratio. Within the training set, the Boruta algorithm (Supplementary Figure S1A) combined with LASSO regression (Supplementary Figures S1B,C) was employed to select prognostic variables from laboratory tests, vital signs, and demographic characteristics. This approach identified eight core predictive features: PNI, RDW, BUN, LDH, Phosphate, TB, HALP score, and GNRI (Supplementary Figure S1D). Based on these selected variables, six machine learning models were constructed. In the training set, the Random Forest (RF) model demonstrated the best discriminative ability, achieving an AUROC of 0.807, an accuracy of 0.800, and a specificity of 0.994. The AUROC values for the other models were as follows: Multilayer Perceptron (MLP): 0.716; Decision Tree: 0.716; Support Vector Machine (SVM): 0.665; Naive Bayes (NB): 0.617; and Logistic Regression: 0.674 (Table 4; Figure 3). In the testing set, the RF model maintained superior performance with an AUROC of 0.754, despite a slight decrease from the training set. This remained higher than the other models: MLP (AUROC = 0.752), Decision Tree (AUROC = 0.697), SVM (AUROC = 0.710), NB (AUROC = 0.716), and Logistic Regression (AUROC = 0.685) (Table 5; Figure 4). Decision curve analysis indicated that, across a range of clinically reasonable threshold probabilities, the RF model provided a superior net benefit compared to “treat-all” or “treat-none” strategies. Calibration curves demonstrated good agreement between the predicted probabilities and observed outcomes for the RF model in the testing set. Collectively, these findings support the Random Forest model as a preferred tool to assist clinical decision-making in this patient population.

**Table 4:** Performance metrics of six machine learning models on the training set.

Model	Accuracy	Recall (Sensitivity)	F1-score	MCC	AUROC	Precision	Specificity
MLP	0.629	0.663	0.455	0.240	0.716	0.346	0.619
RF	0.800	0.166	0.280	0.332	0.807	0.892	0.994
NB	0.667	0.442	0.383	0.163	0.617	0.337	0.735
SVM	0.775	0.040	0.077	0.160	0.665	0.889	0.998
Decision Tree	0.792	0.176	0.283	0.286	0.716	0.729	0.980
Logistic	0.771	0.085	0.148	0.150	0.674	0.567	0.980

The values in the table represent evaluation results on the training set. MLP, Multilayer Perceptron; RF, Random Forest; NB, Naïve Bayes; SVM, Support Vector Machine; Decision Tree, Decision Tree; Logistic, Logistic Regression. Accuracy, proportion of correct predictions; Recall (Sensitivity), true positive rate; F1-score, harmonic mean of precision and recall; MCC, Matthews correlation coefficient; AUROC, area under the receiver operating characteristic curve; Precision, positive predictive value; Specificity, true negative rate.

**Table 5:** Performance metrics of six machine learning models on the test set.

Model	Accuracy	Recall (Sensitivity)	F1-score	MCC	AUROC	Precision	Specificity
MLP	0.669	0.724	0.476	0.311	0.752	0.355	0.655
SVM	0.790	0.039	0.072	0.076	0.710	0.429	0.986
NB	0.721	0.566	0.457	0.289	0.716	0.384	0.762
RF	0.795	0.118	0.194	0.175	0.754	0.529	0.972
Logistic	0.779	0.105	0.165	0.105	0.685	0.381	0.955
Decision Tree	0.806	0.224	0.324	0.274	0.697	0.586	0.959

The values in the table represent evaluation results on the training set. MLP, Multilayer Perceptron; RF, Random Forest; NB, Naïve Bayes; SVM, Support Vector Machine; Decision Tree, Decision Tree; Logistic, Logistic Regression. Accuracy, proportion of correct predictions; Recall (Sensitivity), true positive rate; F1-score, harmonic mean of precision and recall; MCC, Matthews correlation coefficient; AUROC, area under the receiver operating characteristic curve; Precision, positive predictive value; Specificity, true negative rate.

### Five fold cross validation and external validation queue validation

**Figure 5:**
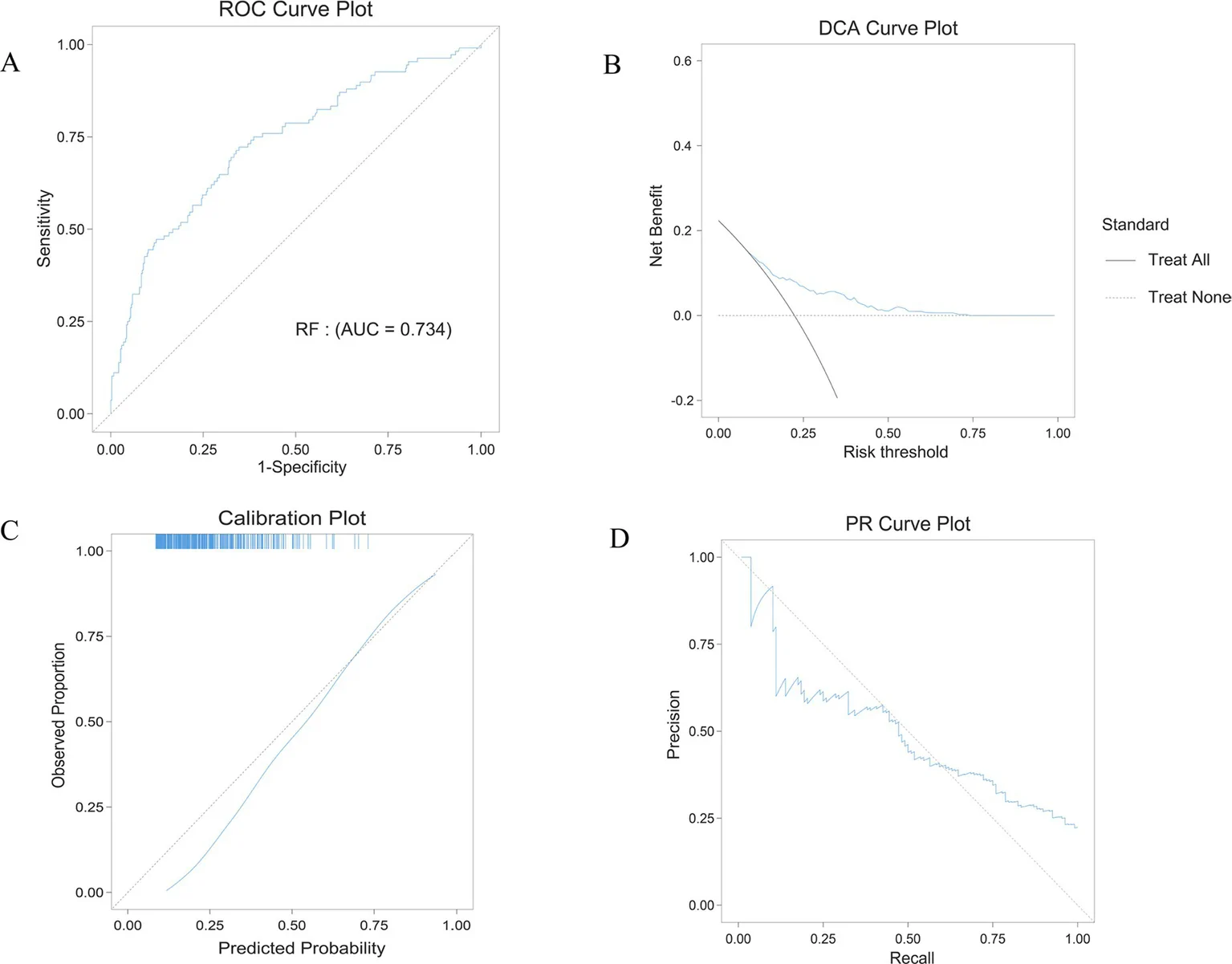
Performance of the RF prediction model in the external validation cohort. **(A)** Receiver operating characteristic (ROC) curve. The area under the curve (AUC) demonstrates the model’s discriminative ability in the external cohort. **(B)** Decision curve analysis (DCA). The net benefit of the model across a range of threshold probabilities is plotted, compared to the strategies of “treat all” and “treat none”. **(C)** Calibration curve. The plot compares the predicted probabilities against the observed event frequencies in the external cohort. The Brier score quantifies the overall calibration error. **(D)** Precision-recall (PR) curve.

To further evaluate the stability of the Random Forest (RF) model, five-fold cross-validation was performed on the training set, and its generalizability was concurrently assessed using an independent external validation cohort. The five-fold cross-validation results (Supplementary Table S2; Supplementary Figure S2) demonstrated stable discriminative performance of the RF model across the five iterations, with a mean AUC of 0.817 (range: 0.813–0.819) and a mean accuracy of 0.808. Although the mean recall (sensitivity) was relatively low at 0.197, the model maintained a very high mean specificity of 0.993 and a high mean precision of 0.895. This pattern suggests a conservative approach to identifying high-risk patients, effectively minimizing false positives. Calibration error remained low across all folds, indicating good agreement between predicted probabilities and observed risk. Decision curve analysis further confirmed that the model provided a positive clinical net benefit across a range of clinically relevant threshold probabilities. The robust generalizability of the RF model was also confirmed in the independent external validation cohort (Figure 5). The model maintained a discriminative ability comparable to that observed in the internal validation, achieving an AUC of 0.734. Calibration curves demonstrated reliable predictions, with good concordance between predicted probabilities and observed outcomes. Decision curve analysis indicated that, across a range of clinically reasonable threshold probabilities, the model offered a net benefit superior to both “treat-all” and “treat-none” strategies.

### Interpretability analysis

**Figure 6:**
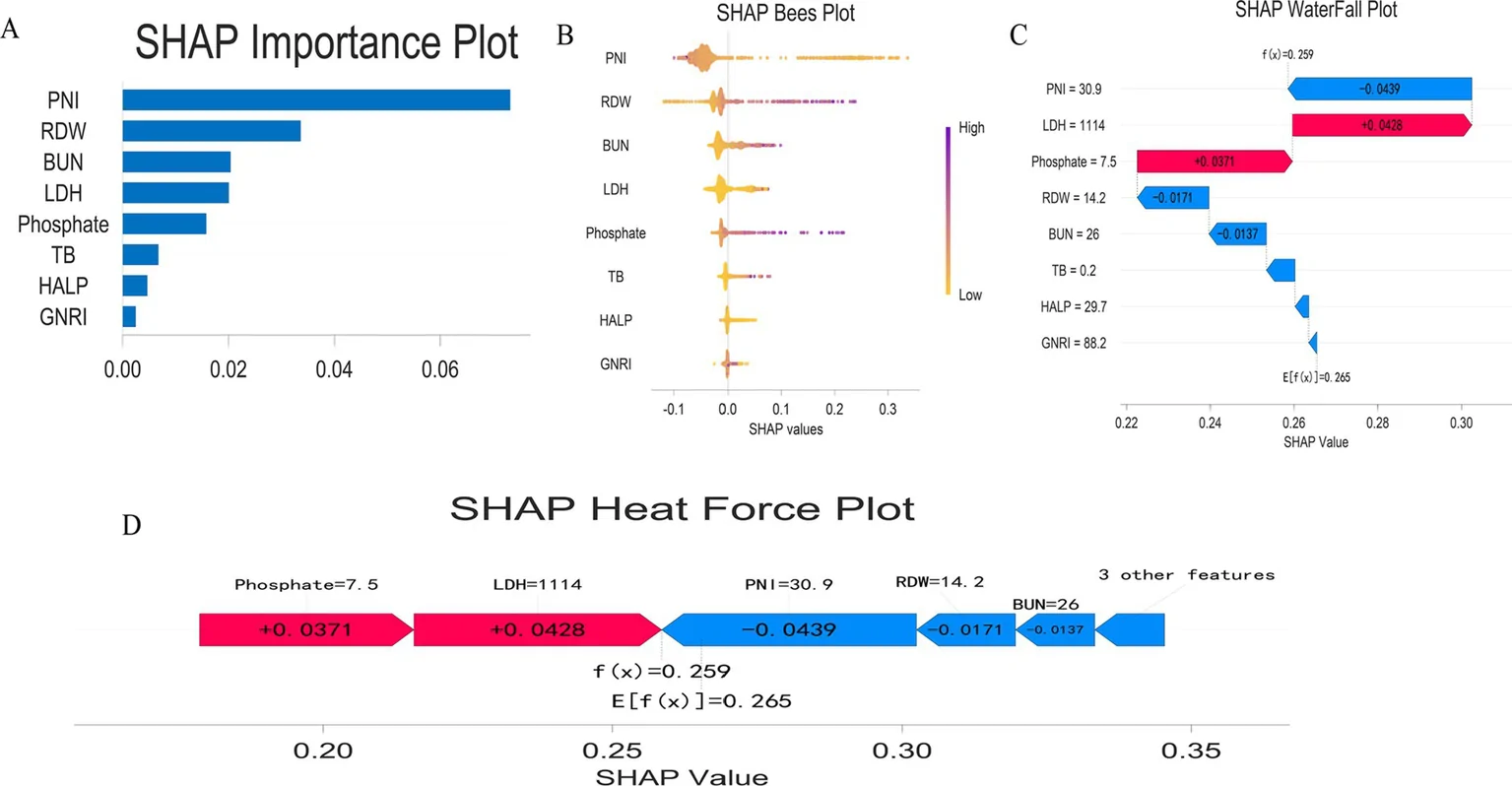
Interpretability analysis of the optimal prediction model using SHapley Additive Explanations (SHAP). **(A)** Global feature importance ranking, identifying PNI as the most influential predictor. **(B)** Beeswarm plot showing feature impact direction: Lower PNI, HALP, GNRI (blue) and higher RDW, BUN, LDH, Phosphate, TB (red) are associated with increased mortality risk. **(C)** Waterfall plot for a representative high-risk patient (predicted mortality: 25.9%), demonstrating that low PNI (30.9) and high LDH (1114) were the primary risk drivers. **(D)** Force plot providing an alternative visualization of the same individual predict.

SHAP analysis was employed to interpret the Random Forest model predictions (Figure 6). The feature importance ranking (Figures 6A,B) showed that PNI was the most significant contributor, followed by RDW, BUN, LDH, Phosphate, TB, HALP, and GNRI. Lower values of PNI, HALP, and GNRI were associated with higher predicted mortality risk, whereas higher values of RDW, BUN, LDH, Phosphate, and TB also increased risk (Figure 6B). An individual prediction decomposition (Figure 6D) illustrated that low PNI and elevated LDH were the primary drivers of increased risk in a representative patient. Overall, the typical high-risk patient profile is characterized by low PNI, elevated BUN, elevated LDH, elevated RDW, along with low HALP, low GNRI, and elevated Phosphate. These findings enhance model transparency and provide quantitative guidance for early identification of high-risk patients.

## Discussion

This study focused on elderly patients (≥65 years), a population in whom malnutrition is particularly prevalent and prognostically important. This multicenter retrospective cohort study systematically evaluated the associations of three composite nutritional indices—PNI, HALP score, and GNRI—with 28-day all-cause mortality in elderly patients with severe respiratory failure, and further developed a machine learning-based predictive model. The findings demonstrated that all three nutritional indices were independent predictors of short-term mortality risk in patients with respiratory failure, exhibiting significant linear dose–response relationships. The RF model, constructed using eight core variables selected by the Boruta algorithm combined with LASSO regression (PNI, RDW, BUN, LDH, Phosphate, TB, HALP score, and GNRI), outperformed other models in both the internal training set (AUC = 0.807) and internal testing set (AUC = 0.752), demonstrating favorable discriminative ability. However, the decline in AUC from the internal test set (0.754) to the external validation set (0.734) warrants consideration. The two cohorts differed geographically (US vs. China), temporally (2008–2019 vs. 2020–2025, with the external cohort overlapping the COVID-19 pandemic), and in diagnostic criteria and clinical practices. These factors likely contributed to the modest AUC drop. Nevertheless, an AUC of 0.734 in an independent cross-continental cohort remains acceptable, and the model maintained good calibration and positive net benefit (Figure 5), supporting its clinical generalizability. SHAP interpretability analysis revealed PNI as the most important driver of model predictions, with the typical profile of high-risk patients characterized by low PNI, elevated BUN, elevated LDH, and elevated RDW.

This study demonstrated that the three nutritional indices—PNI, HALP score, and GNRI—were independently associated with 28-day mortality risk in patients with respiratory failure, exhibiting a clear risk gradient whether analyzed as continuous or categorical variables. This finding corroborates previous research in related fields, including chronic obstructive pulmonary disease, pneumonia, and acute respiratory distress syndrome (29–31). Notably, after full adjustment for multiple confounders, patients with severe malnutrition still exhibited a 33–49% higher mortality risk compared to those with normal nutritional status, suggesting that the impact of nutritional status on prognosis is independent of disease severity and organ dysfunction. The underlying pathophysiological mechanisms may involve several aspects. First, malnutrition directly contributes to respiratory muscle wasting and dysfunction, particularly affecting the diaphragm, which impairs ventilatory drive, prolongs mechanical ventilation, and increases the risk of weaning failure (32). Second, hypoalbuminemia not only reflects depleted nutritional reserves but is also closely associated with capillary leakage, reduced colloid osmotic pressure, and exacerbated pulmonary edema (33). Furthermore, lymphocytopenia and suppressed immune function render patients susceptible to nosocomial infections, which can further amplify inflammatory responses and tissue injury (34). As a composite index integrating albumin and lymphocyte count, the PNI captures the two core dimensions of nutritional reserve and immune status simultaneously, which may explain its emergence as the most important feature among all predictors. Although PNI, HALP score, and GNRI have all been validated as effective prognostic tools, their emphases differ slightly. The GNRI, which combines albumin and body mass index, offers unique advantages for nutritional risk assessment in elderly populations, particularly in critically ill patients for whom recent weight changes may be difficult to ascertain accurately (35). The HALP score, incorporating hemoglobin, albumin, lymphocytes, and platelets, provides a comprehensive reflection of nutritional status, inflammatory burden, and hematopoietic function, and has demonstrated favorable predictive value in patients with sepsis and malignancies (26, 36, 37). In this study, the superior importance of PNI over HALP score and GNRI may be attributable to its direct reflection of the core nutrition-immunity imbalance. This finding suggests that, in patients with respiratory failure, the interplay between immune status and nutritional reserve may carry greater prognostic significance than individual nutritional markers alone.

The combination of the Boruta algorithm and LASSO regression identified eight core features from multidimensional clinical variables, an approach that balanced objective feature selection with model parsimony. The Random Forest model achieved AUCs of 0.807, 0.754, and 0.734 in the training, testing, and external validation sets, respectively, demonstrating robust discriminative ability and stable generalizability. During five-fold cross-validation, the model’s AUC remained stable within the range of 0.813–0.819, and calibration curves alongside decision curve analysis further confirmed its predictive reliability and clinical net benefit. These findings support the model’s potential utility in real-world clinical practice, although its low sensitivity precludes its use as a sole screening instrument. Utilizing the SHAP method, we elucidated the feature importance ranking and the direction of each variable’s influence, identifying decreased PNI and elevated BUN and LDH as the core drivers of high-risk predictions. This finding not only aligns closely with underlying pathophysiological mechanisms but also provides a quantitative basis for individualized risk assessment. For instance, in a patient with respiratory failure presenting with markedly decreased PNI accompanied by elevated BUN and LDH, clinicians could be prompted by the model to initiate early enhanced nutritional support and organ function preservation strategies, thereby establishing a closed-loop pathway from prediction to intervention. The high-risk profile identified through SHAP analysis—characterized by low PNI, elevated BUN, and elevated LDH—offers an intuitive anchor for hypothesis generation, although its translation into clinical decision-making requires further validation and sensitivity optimization.

It should be noted that SHAP analysis provides a measure of relative feature importance within a specific machine learning model, not a causal relationship. The identification of PNI as the most important predictor in this study should be interpreted with caution in the following context. First, the dominance of PNI reflects its contribution to the predictive model rather than proven pathophysiological superiority. Second, the three nutritional indices (PNI, HALP, GNRI) share inherent correlations (e.g., albumin, lymphocytes), and LASSO regularization combined with random forest may favor more parsimonious variables (i.e., PNI) when distributing predictive information among redundant features. Third, SHAP importance rankings depend on the model structure and training data, and may vary across different populations or modeling strategies. Therefore, the high importance of PNI should be interpreted as a strong predictive contribution within our model, not as definitive evidence of biological superiority over other nutritional indices. Future studies should externally validate the comparative performance of different nutritional indices and explore the development of an optimized score integrating multidimensional information.

Although this study provides an objective and quantifiable tool for early risk stratification in elderly patients with severe respiratory failure, several limitations should be acknowledged. First, despite the multicenter design, the internal cohort was derived from a US public database (MIMIC-IV) and the external cohort from a single Chinese center; differences in population characteristics and treatment patterns may affect model generalizability. Second, the retrospective design is subject to selection bias and information bias, and some potential confounders (e.g., specific nutritional support protocols, medication adherence) could not be included. Moreover, the model exhibited low recall (sensitivity) in external validation (0.118–0.197 in cross-validation), indicating that it misclassifies approximately 80% of patients who died. This limitation severely restricts its current clinical utility as a screening or early warning tool. The model’s primary value at this stage lies in its high specificity, which reliably identifies the most clinically obvious high-risk cases, rather than serving as a comprehensive risk stratification instrument for all patients. Third, several limitations related to nutritional assessment should be noted: nutritional indices were based only on admission measurements and did not capture dynamic changes during hospitalization; variables such as ideal body weight rely on height and weight measurements, which may introduce measurement bias in critically ill patients; and the three indices share common components (albumin, lymphocytes), limiting the diversity of captured information. We also did not include direct inflammatory markers (e.g., CRP) or metabolic parameters (e.g., total cholesterol), although these were omitted for pragmatic reasons—they are not universally available at ICU admission. Fourth, the diagnostic criteria for respiratory failure differed between the two cohorts (ICD codes plus blood gas in the internal cohort vs. a comprehensive clinical definition in the external cohort), which could introduce selection bias. Nonetheless, the consistent results across both cohorts support the robustness of our findings. Future prospective multicenter studies are needed to further validate the model’s generalizability and clinical effectiveness, and to develop time-dependent prediction models incorporating dynamic nutritional changes.

Lastly, and most importantly, the low sensitivity of our Random Forest model represents a fundamental limitation that warrants further discussion. We empirically explored two strategies to improve recall: class-weighted training and threshold optimization based on the precision-recall curve. As shown in Supplementary Table S7, both approaches improved sensitivity (e.g., from 0.118 to 0.235 in the test set for class-weighted training, and to 0.285 at a 0.3 cutoff), but at the cost of substantially reduced specificity (from 0.972 to 0.928 and 0.897, respectively). This sensitivity-specificity trade-off is inherent to the current feature set and reflects the difficulty of distinguishing patients who will die from those who will survive using only admission-based nutritional indices and routine laboratory parameters. Importantly, while improving recall is desirable for a screening tool, the resulting increase in false positives would lead to unnecessary interventions, resource utilization, and potential patient harm. We also considered SMOTE-based oversampling but did not adopt it, as synthetic samples may not reflect physiologically plausible patient profiles in critically ill populations, and oversampling risks inflating performance estimates while compromising external generalizability. We believe that the optimal balance between sensitivity and specificity can only be determined through prospective evaluation in real-world settings, where the relative harms of false negatives versus false positives can be weighed against tangible clinical outcomes. Until such studies are conducted, our model should be viewed as a proof-of-concept tool rather than a clinically ready instrument.

## Conclusion

This study confirms that PNI, HALP score, and GNRI are independent predictors of 28-day all-cause mortality in elderly patients with severe respiratory failure. Within the machine learning-based predictive model incorporating multiple indicators, PNI showed the highest feature importance in our SHAP analysis, reflecting its strong contribution to the predictive model. SHAP interpretability analysis identified the typical profile of high-risk patients characterized by low PNI, elevated BUN, and elevated LDH. This interpretable model provides a quantitative tool for risk identification. However, its low sensitivity limits its current clinical readiness; future work should focus on optimizing sensitivity—for example through threshold adjustment or ensemble strategies—followed by prospective validation of clinical effectiveness.

## Data Availability

The datasets presented in this study can be found in online repositories. The names of the repository/repositories and accession number(s) can be found in the article/Supplementary material.
