# Supplementary material for: The predictive value of malnutrition on the prognosis of severe respiratory failure in elderly patients: a multicenter retrospective study based on interpretable machine learning
Source: Front Nutr. 2026 Aug 7;13:1833732. doi: 10.3389/fnut.2026.1833732 (PMC13493236; doi:10.3389/fnut.2026.1833732)
Supplement: Supplementary file 1 [file Data_sheet_1.pdf]

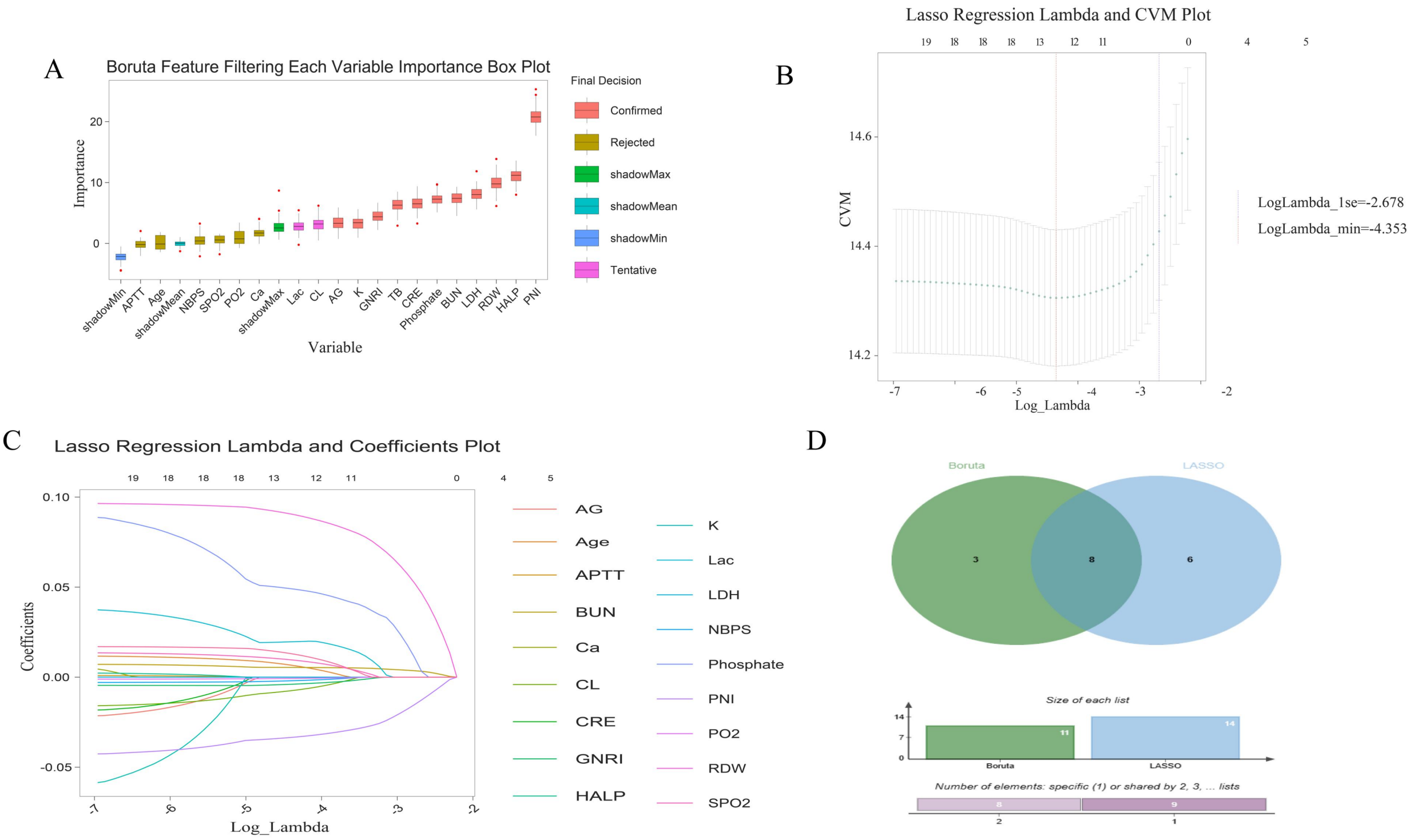

Figure S1: The screening process of feature variables.

Note: (A):Boruta feature ranking algorithm, The box plots depict the distribution of Z-scores (a measure of importance) for each original variable (colored boxplots) across multiple iterations, compared against the maximum importance of shuffled “shadow” features (blue and green boxplots). Variables are ranked by their median Z-score. Features whose importance (colored boxes) consistently exceeds that of the best shadow feature (the green “shadowMax” boxplot) are deemed ‘confirmed’ predictors (highlighted in the plot). (B) LASSO coefficient profiles of the candidate prognostic variables. Each curve represents the coefficient path of a variable as the penalty parameter (λ) increases.(C) Ten-fold cross-validation for tuning parameter (λ) selection in the LASSO model. The left vertical dashed line (lambda.min) is drawn at the value of λ that gives the minimum mean cross-validated error. The right vertical dashed line (lambda.1se) is drawn at the largest value of λ such that the error is within one standard error of the minimum, which was used for feature selection.(D)The Venn diagram shows the overlap of candidate predictor variables identified as important in two feature selection methods.

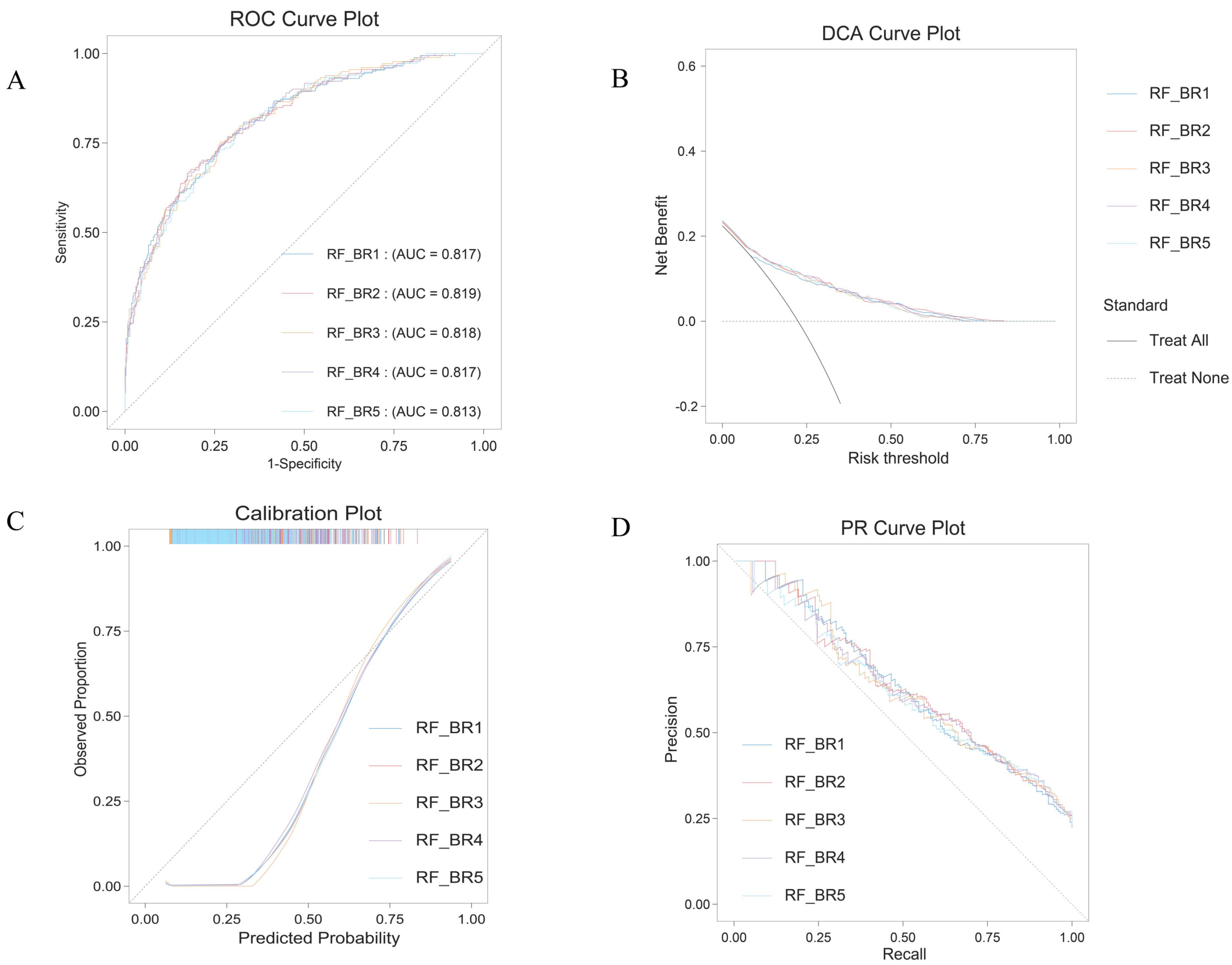

Figure S2: Performance evaluation of six machine learning prediction models on the testing set.

Note: (A) Receiver operating characteristic (ROC) curves. The area under the curve (AUC) with 95% confidence intervals is shown for each model. (B) Decision curve analysis (DCA). The net benefit across a range of threshold probabilities is plotted. The gray solid line represents the strategy of “treating all” patients, and the gray dashed line represents “treating none”. A model curve above these lines indicates clinical utility within that probability range. (C) Calibration curves. The plots compare predicted probabilities against observed event frequencies. The dotted diagonal line represents perfect calibration. (D) Precision-recall (PR) curves. Models: Random Forest (RF), K-Nearest Neighbors (KNN), Logistic Regression (Logistic), Multi-layer Perceptron (MLP), Decision Tree (DT), and Naïve Bayes (NB).

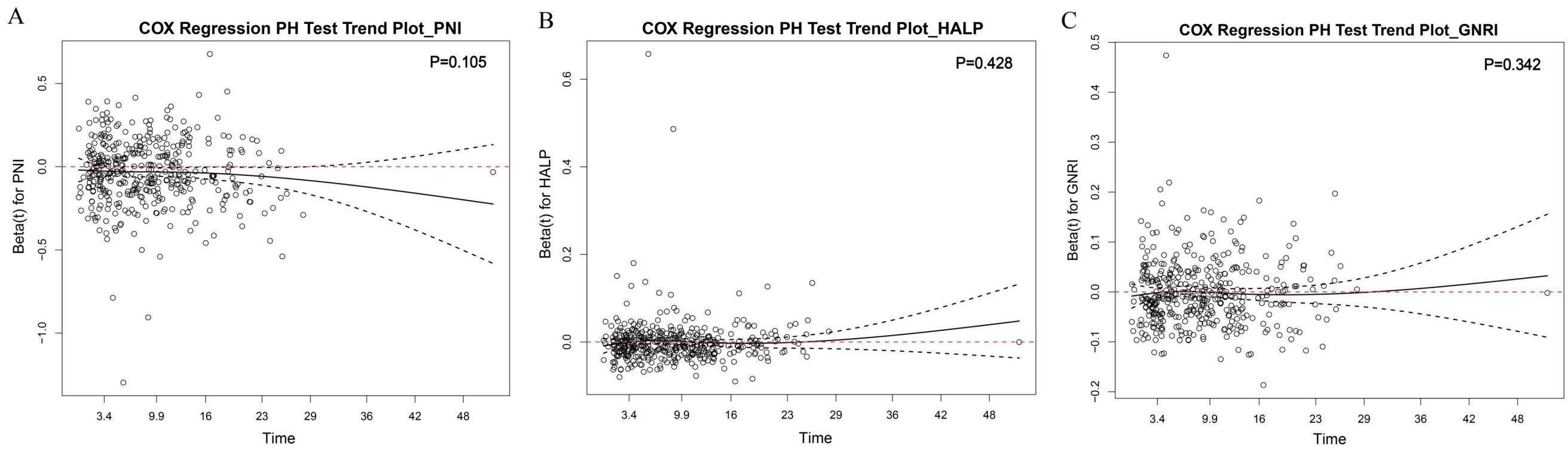

Figure S3: COX Regression PH Test Trend Plot.

Table S1: Baseline characteristics of the overall study population and stratified by 28-day ICU survival status in the external verification cohort.

Variable	ALL *N=592*	Survivor *N=400*	No-survivor *N=192*	P-value
PNI	35.3 (7.54)	36.6 (7.24)	32.5 (7.42)	<0.001
PNI_group:				<0.001
moderate	88 (14.9%)	67 (16.8%)	21 (10.9%)	
normal	205 (34.6%)	158 (39.5%)	47 (24.5%)	
serious	299 (50.5%)	175 (43.8%)	124 (64.6%)	
HALP	23.7 (28.3)	26.3 (31.6)	18.1 (18.5)	<0.001
HALP_group:				<0.001
moderate	198 (33.4%)	132 (33.0%)	66 (34.4%)	
normal	197 (33.3%)	155 (38.8%)	42 (21.9%)	
serious	197 (33.3%)	113 (28.2%)	84 (43.8%)	
GNRI	98.9 (17.9)	101 (17.7)	94.7 (17.9)	<0.001
GNRI_group:				<0.001
low	92 (15.5%)	71 (17.8%)	21 (10.9%)	
moderate	134 (22.6%)	84 (21.0%)	50 (26.0%)	
normal	283 (47.8%)	206 (51.5%)	77 (40.1%)	

Variable	ALL<br>*N=592*	Survivor<br>*N=400*	No-survivor<br>*N=192*	P-value
serious	83 (14.0%)	39 (9.75%)	44 (22.9%)	
Age	76.1 (7.55)	75.7 (7.41)	76.9 (7.80)	0.072
Gender:				0.477
F	273 (46.1%)	189 (47.2%)	84 (43.8%)	
M	319 (53.9%)	211 (52.8%)	108 (56.2%)	
BMI(Kg/m^2)	29.2 (7.66)	29.5 (7.83)	28.4 (7.26)	0.091
hypertension:				0.265
No	378 (63.9%)	262 (65.5%)	116 (60.4%)	
Yes	214 (36.1%)	138 (34.5%)	76 (39.6%)	
AKI:				0.018
No	198 (33.4%)	147 (36.8%)	51 (26.6%)	
Yes	394 (66.6%)	253 (63.2%)	141 (73.4%)	
CKD:				0.298
No	407 (68.8%)	269 (67.2%)	138 (71.9%)	
Yes	185 (31.2%)	131 (32.8%)	54 (28.1%)	
DM:				0.885

Variable	ALL *N=592*	Survivor *N=400*	No-survivor *N=192*	P-value
No	374 (63.2%)	254 (63.5%)	120 (62.5%)	
Yes	218 (36.8%)	146 (36.5%)	72 (37.5%)	
HLD:				0.155
No	288 (48.6%)	186 (46.5%)	102 (53.1%)	
Yes	304 (51.4%)	214 (53.5%)	90 (46.9%)	
CB:				0.293
No	452 (76.4%)	311 (77.8%)	141 (73.4%)	
Yes	140 (23.6%)	89 (22.2%)	51 (26.6%)	
HF:				0.223
No	307 (51.9%)	200 (50.0%)	107 (55.7%)	
Yes	285 (48.1%)	200 (50.0%)	85 (44.3%)	
IHD:				0.647
No	308 (52.0%)	205 (51.2%)	103 (53.6%)	
Yes	284 (48.0%)	195 (48.8%)	89 (46.4%)	
COPD:				0.091
No	423 (71.5%)	295 (73.8%)	128 (66.7%)	

Variable	ALL<br>*N=592*	Survivor<br>*N=400*	No-survivor<br>*N=192*	P-value
Yes	169 (28.5%)	105 (26.2%)	64 (33.3%)	
Sofa	7.23 (3.93)	6.72 (3.73)	8.29 (4.15)	<0.001
APSIII	56.7 (23.4)	52.9 (21.4)	64.7 (25.3)	<0.001
SAPSII	47.4 (14.4)	45.1 (13.5)	52.3 (15.0)	<0.001
OASIS	38.1 (8.70)	36.8 (8.37)	40.8 (8.78)	<0.001
Charlson	6.66 (2.43)	6.61 (2.34)	6.77 (2.61)	0.483
APACHEII	23.0 (7.69)	22.0 (7.51)	24.9 (7.72)	<0.001
HR(beats/min)	89.3 (20.7)	88.2 (20.4)	91.6 (21.2)	0.068
NBPS(mmHg)	122 (25.3)	124 (26.0)	118 (23.4)	0.007
NBPD(mmHg)	70.5 (20.8)	70.8 (20.9)	70.0 (20.7)	0.669
NBPM(mmHg)	84.5 (20.5)	85.2 (21.0)	83.1 (19.5)	0.243
RR(insp/min)	97.7 (36.1)	98.4 (43.8)	96.2 (4.73)	0.321
SPO2(%)	21.0 (6.83)	20.7 (6.68)	21.5 (7.13)	0.188
Temperaturef(°F)	98.2 (2.95)	98.2 (3.37)	98.1 (1.79)	0.573
Lymphocyte_count(%)	1.16 (0.91)	1.25 (0.97)	0.96 (0.76)	<0.001
HCT(%)	32.7 (7.69)	33.1 (7.55)	31.9 (7.94)	0.085

Variable	ALL<br>*N=592*	Survivor<br>*N=400*	No-survivor<br>*N=192*	P-value
Hb(g/dL)	105 (25.6)	106 (25.5)	101 (25.7)	0.028
PLT(K/μL)	196 (106)	196 (101)	195 (117)	0.922
RDW(%)	15.4 (2.46)	15.2 (2.36)	16.0 (2.60)	0.001
RBC(m/uL)	3.53 (0.87)	3.58 (0.84)	3.43 (0.92)	0.063
WBC(K/μL)	13.7 (9.78)	13.7 (10.7)	13.6 (7.63)	0.917
Neutrophil_count(%)	11.1 (7.13)	10.7 (6.78)	12.1 (7.74)	0.028
ALB(g/L)	29.5 (6.03)	30.4 (5.70)	27.7 (6.32)	<0.001
AG(mEq/L)	14.9 (4.88)	14.8 (4.64)	15.2 (5.36)	0.440
Ca(mg/dL)	8.35 (0.79)	8.38 (0.74)	8.28 (0.89)	0.157
CL(mEq/L)	103 (6.63)	103 (6.64)	103 (6.64)	0.758
Glu(mg/dL)	160 (73.8)	155 (67.9)	171 (84.0)	0.026
K(mEq/L)	4.35 (0.80)	4.32 (0.76)	4.42 (0.89)	0.146
Na(mEq/L)	138 (5.63)	139 (5.47)	138 (5.97)	0.506
TCO2(mEq/L)	24.3 (6.28)	24.7 (5.90)	23.6 (6.96)	0.065
Lac(mmol/L)	2.49 (2.19)	2.36 (2.03)	2.75 (2.47)	0.057
PCO2(mmHg)	45.0 (13.5)	44.7 (13.0)	45.5 (14.5)	0.549

Variable	ALL<br>*N=592*	Survivor<br>*N=400*	No-survivor<br>*N=192*	P-value
PH(pH units)	7.34 (0.11)	7.35 (0.11)	7.31 (0.12)	0.002
PO2(mmHg)	115 (102)	119 (104)	109 (96.9)	0.256
INR(ratio)	1.57 (0.85)	1.52 (0.83)	1.68 (0.89)	0.033
PT(seconds)	17.1 (9.41)	16.5 (9.11)	18.3 (9.90)	0.030
APTT(seconds)	41.5 (30.4)	40.7 (30.1)	43.1 (30.9)	0.374
ALT(IU/L)	126 (384)	125 (416)	128 (307)	0.916
AST(IU/L)	165 (464)	169 (523)	155 (306)	0.671
TB(mg/dL)	1.11 (2.06)	0.98 (1.49)	1.38 (2.88)	0.077
CRE(mg/dL)	1.63 (1.32)	1.59 (1.34)	1.71 (1.28)	0.277
BUN(mg/dL)	34.6 (25.3)	31.9 (23.2)	40.1 (28.5)	0.001
LDH(U/L)	499 (1049)	450 (731)	600 (1507)	0.193
BE(mEq/L)	-2.09 (6.16)	-1.61 (5.68)	-3.08 (6.97)	0.011
Mg(mg/dL)	2.11 (1.16)	2.15 (1.38)	2.04 (0.41)	0.128
Phosphate	4.10 (1.56)	3.94 (1.44)	4.42 (1.73)	0.001
Monocyte_count(%)	0.94 (0.84)	0.91 (0.80)	1.00 (0.91)	0.260
Ventilation:				0.046

Variable	ALL *N=592*	Survivor *N=400*	No-survivor *N=192*	P-value
No	27 (4.56%)	13 (3.25%)	14 (7.29%)	
Yes	565 (95.4%)	387 (96.8%)	178 (92.7%)	
CRRT:				<0.001
No	499 (84.3%)	353 (88.2%)	146 (76.0%)	
Yes	93 (15.7%)	47 (11.8%)	46 (24.0%)	
Sa:				0.055
No	55 (9.29%)	44 (11.0%)	11 (5.73%)	
Yes	537 (90.7%)	356 (89.0%)	181 (94.3%)	
VP:				<0.001
No	102 (17.2%)	85 (21.2%)	17 (8.85%)	
Yes	490 (82.8%)	315 (78.8%)	175 (91.1%)	
GC:				0.208
No	341 (57.6%)	238 (59.5%)	103 (53.6%)	
Yes	251 (42.4%)	162 (40.5%)	89 (46.4%)	
AHT:				0.134
No	68 (11.5%)	40 (10.0%)	28 (14.6%)	

Variable	ALL *N=592*	Survivor *N=400*	No-survivor *N=192*	P-value
Yes	524 (88.5%)	360 (90.0%)	164 (85.4%)	
ABX:				1.000
No	21 (3.55%)	14 (3.50%)	7 (3.65%)	
Yes	571 (96.5%)	386 (96.5%)	185 (96.4%)	
Survival_time	21.6 (9.75)	27.9 (1.64)	8.55 (5.90)	<0.001

Note: Data are presented as mean (standard deviation) for continuous variables and number (percentage) for categorical variables. Between-group comparisons were performed using the Chi-square test for categorical variables and Student's t-test or Mann-Whitney U test for continuous variables, as appropriate. Abbreviations: Prognostic Nutritional Index (PNI); Hemoglobin, Albumin, Lymphocyte, and Platelet score (HALP); Geriatric Nutritional Risk Index (GNRI); Body Mass Index (BMI); Acute Kidney Injury (AKI); Chronic Kidney Disease (CKD); Diabetes Mellitus (DM); Hyperlipidemia (HLD); Chronic bronchitis (CB); Heart Failure (HF); Ischemic Heart Disease (IHD); Chronic Obstructive Pulmonary Disease (COPD); Sequential Organ Failure Assessment (SOFA); Acute Physiology Score III (APS III); Simplified Acute Physiology Score II (SAPS II); Oxford Acute Severity of Illness Score (OASIS); Charlson Comorbidity Index (Charlson); Acute Physiology and Chronic Health Evaluation II (APACHE II); Heart Rate (HR); Non-Invasive Blood Pressure Systolic/Diastolic/Mean (NBPS/NBPD/NBPM); Respiratory Rate (RR); Oxygen Saturation (SpO_2); Temperature in Fahrenheit (Temperaturef); Lymphocyte count (Lymphocyte_count); Hematocrit (HCT); Hemoglobin (Hb); Platelet count (PLT); Red Cell Distribution Width (RDW); Red Blood Cell count (RBC); White Blood Cell count (WBC); Neutrophil count (Neutrophil_count); Albumin (ALB); Anion Gap (AG); Total Calcium (Ca); Chloride (CL); Glucose (Glu); Potassium (K); Sodium (Na); Total Carbon Dioxide (TCO_2); Lactate (Lac); Partial Pressure of Carbon Dioxide (PCO_2); Potential of Hydrogen (PH); Partial Pressure of Oxygen (PO_2); International Normalized Ratio (INR); Prothrombin Time (PT); Activated Partial Thromboplastin Time (APTT); Alanine Aminotransferase (ALT); Aspartate Aminotransferase (AST); Total Bilirubin (TB); Creatinine (CRE); Blood Urea Nitrogen (BUN); Lactate Dehydrogenase (LDH); Base Excess (BE); Magnesium (Mg); Phosphate (Phosphate); Monocyte count (Monocyte_count);

Continuous Renal Replacement Therapy (CRRT); Analgesic and sedative drugs (Sa); Vasopressor (VP); Glucocorticoid (GC); Antihypertensive Therapy (AHT); Antibiotics (ABX).

Table S2. Performance indicators of Random Forest model in five fold cross validation of training cohort

Model	Accuracy	Recall (Sensitivity)	F1- Score	MCC	AUROC	Precision	Specificity
**Fold 1**	0.817	0.209	0.340	0.380	0.817	0.900	0.993
**Fold 2**	0.812	0.223	0.357	0.387	0.819	0.889	0.991
**Fold 3**	0.807	0.180	0.302	0.362	0.818	0.941	0.997
**Fold 4**	0.806	0.210	0.338	0.365	0.817	0.864	0.990
**Fold 5**	0.797	0.165	0.278	0.327	0.813	0.882	0.993
**Mean**	0.808	0.197	0.323	0.364	0.817	0.895	0.993

Note: The values in the table are all results of five fold cross validation. Fold 1~5 represents 50% off data, and Mean is the average value. Accuracy: Accuracy; Recall (Sensitivity): Recall rate (sensitivity); F1 score: the harmonic mean of precision and recall; MCC: Matthews correlation coefficient; AUROC: Area under the receiver operating characteristic curve; Precision: accuracy rate; Specificity: specificity.

Table S3: Coherent linear screening (VIF) in each cohort

**Internal MIMIC cohort**		**External verification cohort**	
**Variable Names**	**VIF**	**Variable Names**	**VIF**
Ventilation	1.10	Temperaturef	1.09

Internal MIMIC cohort		External verification cohort	
**Variable Names**	**VIF**	**Variable Names**	**VIF**
GC	1.11	RBC	1.13
HLD	1.11	Ventilation	1.15
NBPS	1.13	APTT	1.17
APTT	1.14	NBPS	1.19
Sa	1.19	LDH	1.19
SPO2	1.20	VP	1.24
VP	1.21	RDW	1.24
AHT	1.25	Sa	1.30
PO2	1.27	AKI	1.34
AKI	1.32	Age	1.37
Ca	1.33	TB	1.37
LDH	1.35	CRRT	1.47
TB	1.41	HALP	1.54
Charlson	1.41	GNRI	1.70
Age	1.43	PNI	2.03
K	1.44	Lac	2.04
RDW	1.48	Phosphate	2.46
CRRT	1.53	OASIS	2.67
GNRI	1.62	Sofa	2.83
HALP	1.72	BUN	2.97
CL	1.72	APACHEII	3.41
PNI	2.28	CRE	3.49
BUN	2.43	SAPSII	4.39
OASIS	2.44	APSIII	4.45

Internal MIMIC cohort		External verification cohort	
**Variable Names**	**VIF**	**Variable Names**	**VIF**
Phosphate	2.64	PH	10.92
Lac	2.77	TCO2	26.96
CRE	2.82	BE	49.45
Sofa	2.84	PT	1061.15
AG	3.42	INR	1062.62
APACHEII	3.57		
SAPSII	4.30		
APSIII	4.55		
RBC	9.29		
PH	10.93		
TCO2	30.30		
Hb	30.46		
HCT	36.96		
BE	54.35		
PT	779.48		
INR	779.97		

Note: Prognostic Nutritional Index (PNI); Geriatric Nutritional Risk Index (GNRI); Hemoglobin, Albumin, Lymphocyte, and Platelet score (HALP); Blood Urea Nitrogen (BUN); Lactate Dehydrogenase (LDH); Total Bilirubin (TB); Red Cell Distribution Width (RDW); Creatinine (CRE); Phosphate (Phosphate); Lactate (Lac); Calcium (Ca); Potassium (K); Chloride (CL); Anion Gap (AG); Potential of Hydrogen (PH); Total Carbon Dioxide (TCO_2); Base Excess (BE); Hemoglobin (Hb); Hematocrit (HCT); Red Blood Cell count (RBC); Prothrombin Time (PT); International Normalized Ratio (INR); Activated Partial Thromboplastin Time (APTT); Age (Age); Sequential Organ Failure Assessment (SOFA); Acute Physiology Score III (APS III); Simplified Acute Physiology Score II (SAPS II); Oxford Acute Severity of Illness Score (OASIS); Acute Physiology and Chronic Health Evaluation II (APACHE II); Charlson Comorbidity Index (Charlson); Non-Invasive Blood Pressure Systolic (NBPS); Oxygen Saturation (SpO_2); Partial Pressure of Oxygen (PO_2); Continuous Renal Replacement Therapy (CRRT); Vasopressor (VP);

Antihypertensive Therapy (AHT); Analgesic and sedative drugs (Sa); Acute Kidney Injury (AKI).

TableS4: Statistical table of missing values for each variable

Variable Names	level	Overall	Missing
N		1385	
Age		74 (65-99)	0
Weight		77.9 (27.3-575)	0
height		170 (137-203)	0
BMI		27.86 (12.287-93.297)	0
SOFA		7 (0-19)	0
APSIII		52 (14-149)	0
SAPSII		45 (18-114)	0
OASIS		38 (16-64)	0
charlson		6 (2-17)	0
APACHEII		22 (5-52)	0
HR		88 (28-179)	1.3
NBPS		119 (39-245)	1.6
NBPD		67 (12-199)	3.5
RR		81 (19-936)	0.2
Spo2		97 (0-963)	0
temperaturef		20 (0-63)	0.8
absolute_lymphocyte_count		0.95 (0.01-24.03)	0
HCT		32.8 (13-55.7)	0.2
HB		104 (41-190)	0
PLT		183 (9-909)	0
RDW		14.8 (11.4-28.4)	0.5
RBC		3.52 (1.16-6.82)	0
WBC		12.3 (0.1-139)	0.7
absolute_neutrophil_count		10.14 (0-70.98)	25.5
HbA1c		5.7 (4-14)	61.7
ALB		30 (9-49)	0
AG		15 (1-47)	0
Ca		8.3 (5-13.6)	0.4
Cl		103 (73-135)	0.1
GLU		144 (30-1260)	5.4

Variable Names	level	Overall	Missing
K		4.3 (2-8.7)	3.1
Na		139 (108-170)	11.8
TCO2		24 (4-58)	0.1
Lac		1.8 (0.3-22)	12.8
free_calcium		1.11 (0.71-1.66)	38.8
Pco2		42 (14-121)	3.9
ph		7.35 (6.87-7.7)	21.5
po2		77 (14-612)	6.9
ddimer		3312 (364-21240)	97
FIB		348 (29-1306)	73
INR		1.3 (0.9-13.1)	3.9
PT		14.3 (10-150)	4.8
APTT		31.1 (17.8-150)	5.3
thrombin		18.2 (12.7-150)	98.6
HDL		45 (15-117)	65.9
LDL		85.5 (7-321)	66.5
TC		156.5 (37-388)	65.4
TG		117 (16-2786)	54.2
ALT		26 (5-7392)	4.7
AST		42 (5-27004)	3.7
DBIL		1.15 (0.4-18.2)	89
IBIL		0.7 (0.2-15.3)	88.8
TB		0.6 (0.1-31.9)	3.7
CRE		1.2 (0-17.3)	0
URE		27 (5-212)	0.1
UC		4.6 (0.8-14.4)	92.7
CK		171.5 (10-72670)	41.2
CK-MB		4 (1-411)	52.3
LDH		324 (85-19980)	0
NT-pro-BNP		2464 (25-63198)	90.5
troponin_t		0.08 (0-8.16)	73.6
lymphocytes		10.9 (0-100)	34.4
Mg		6.8 (0-46)	34.4

Variable Names	level	Overall	Missing
neutrophils		79 (0-96)	34.4
absolute_monocyte_count		0.8 (0-49.22)	19.5
Phosphate		3.8 (0.4-12.8)	2.5
28day-Survival_time		28 (0.23-28)	0
gender (%)	F	598 (43.18)	0
gender (%)	M	787 (56.82)	
Hypertension (%)	No	601 (43.39)	0
Hypertension (%)	Yes	784 (56.61)	
Race (%)	Other races	601 (43.39)	0
Race (%)	WHITE	784 (56.61)	
AKI (%)	No	461 (33.29)	0
AKI (%)	Yes	924 (66.71)	
CKD (%)	No	942 (68.01)	0
CKD (%)	Yes	443 (31.99)	
Diabetes (%)	No	856 (61.81)	0
Diabetes (%)	Yes	529 (38.19)	
HLD (%)	No	724 (52.27)	0
HLD (%)	Yes	661 (47.73)	
IHD (%)	No	708 (51.12)	0
IHD (%)	Yes	677 (48.88)	
COPD (%)	No	1022 (73.79)	0
COPD (%)	Yes	363 (26.21)	
CB (%)	No	1069 (77.18)	0
CB (%)	Yes	316 (22.82)	
HF (%)	No	739 (53.36)	0
HF (%)	Yes	646 (46.64)	
Sa (%)	No	119 (8.59)	0
Sa (%)	Yes	1266 (91.41)	
Vp (%)	No	246 (17.76)	0
Vp (%)	Yes	1139 (82.24)	
Gc (%)	No	820 (59.21)	0
Gc (%)	Yes	565 (40.79)	
ABX (%)	No	65 (4.69)	0

Variable Names	level	Overall	Missing
ABX (%)	Yes	1320 (95.31)	
AHT (%)	No	166 (11.99)	0
AHT (%)	Yes	1219 (88.01)	
Ventilation (%)	No	62 (4.48)	0
Ventilation (%)	Yes	1323 (95.52)	
CRRT (%)	No	1156 (83.47)	0
CRRT (%)	Yes	229 (16.53)	
Death_28days (%)	0	983 (70.97)	0
Death_28days (%)	1	402 (29.03)	

Table S5: Multi factor Cox regression (modle3) proportional hazards assessment

Variable Name	df	P-value
Age	1	0.086
AKI	1	0.074
HLD	1	0.385
Sofa	1	0.218
APSIII	1	0.609
SAPSII	1	0.215
OASIS	1	0.717
Charlson	1	0.550
APACHEII	1	0.712
NBPS	1	0.642

SPO2	1	0.068
RDW	1	0.045
AG	1	0.608
Ca	1	0.074
CL	1	0.008
K	1	0.203
Lac	1	0.778
PO2	1	0.440
APTT	1	0.176
TB	1	0.129
CRE	1	0.460
BUN	1	0.873
LDH	1	0.420
Phosphate	1	0.219
Ventilation	1	0.006
CRRT	1	0.000
Sa	1	0.012
VP	1	0.016
GC	1	0.457
AHT	1	0.000
PNI	1	0.105
HALP	1	0.428
GNRI	1	0.342
GLOBAL	33	0.188

Note: Prognostic Nutritional Index (PNI); Hemoglobin, Albumin, Lymphocyte, and Platelet score (HALP); Geriatric Nutritional Risk Index (GNRI); Blood Urea Nitrogen (BUN); Lactate Dehydrogenase (LDH); Total Bilirubin (TB); Red Cell Distribution Width (RDW); Creatinine (CRE); Phosphate (Phosphate); Lactate (Lac);

Calcium (Ca); Potassium (K); Chloride (CL); Anion Gap (AG); Partial Pressure of Oxygen (PO_2); Activated Partial Thromboplastin Time (APTT); Age (Age); Acute Kidney Injury (AKI); Hyperlipidemia (HLD); Sequential Organ Failure Assessment (SOFA); Acute Physiology Score III (APS III); Simplified Acute Physiology Score II (SAPS II); Oxford Acute Severity of Illness Score (OASIS); Acute Physiology and Chronic Health Evaluation II (APACHE II); Charlson Comorbidity Index (Charlson); Non-Invasive Blood Pressure Systolic (NBPS); Oxygen Saturation (SpO_2); Continuous Renal Replacement Therapy (CRRT); Vasopressor (VP); Antihypertensive Therapy (AHT); Analgesic and sedative drugs (Sa); Mechanical Ventilation (Ventilation); Glucocorticoids (GC); Global test (GLOBAL).

Table S6: Comparison of baseline characteristics between the internal (MIMIC-IV) and external (Binzhou Medical University Hospital) validation cohorts

**Characteristic**	**Internal cohort (N=1,385)**	**External cohort (N=592)**	**P-value**
Demographics			
Age, years (mean ± SD)	75.7 ± 7.51	76.1 ± 7.55	0.284
Male, n (%)	787 (56.8%)	319 (53.9%)	0.237
BMI, kg/m² (mean ± SD)	29.3 ± 8.10	29.2 ± 7.66	0.797
Race (White), n (%)	784 (56.6%)	—	—
Comorbidities, n (%)			
Hypertension	478 (34.5%)	214 (36.1%)	0.490
AKI	924 (66.7%)	394 (66.6%)	0.943
CKD	443 (32.0%)	185 (31.2%)	0.742
Diabetes mellitus	529 (38.2%)	218 (36.8%)	0.571
HLD	661 (47.7%)	304 (51.4%)	0.140
Chronic bronchitis	316 (22.8%)	140 (23.6%)	0.693
HF	646 (46.6%)	285 (48.1%)	0.546
IHD	677 (48.9%)	284 (48.0%)	0.712
COPD	363 (26.2%)	169 (28.5%)	0.287
Severity scores (mean ± SD)			
SOFA	7.19 ± 3.85	7.23 ± 3.93	0.830
APS III	55.7 ± 22.4	56.7 ± 23.4	0.365

Characteristic	Internal cohort (N=1,385)	External cohort (N=592)	P-value
SAPS II	47.0 ± 13.8	47.4 ± 14.4	0.561
OASIS	37.7 ± 8.52	38.1 ± 8.70	0.341
Charlson Comorbidity Index	6.67 ± 2.45	6.66 ± 2.43	0.933
APACHE II	22.9 ± 7.63	23.0 ± 7.69	0.789
Nutritional indices (mean ± SD)			
PNI	35.6 ± 8.67	35.3 ± 7.54	0.460
PNI_group, n (%)			0.825
Normal	474 (34.2%)	205 (34.6%)	
Moderate	222 (16.0%)	88 (14.9%)	
Severe	689 (49.7%)	299 (50.5%)	
HALP score	25.5 ± 35.5	23.7 ± 28.3	0.263
HALP_group, n (%)			0.847
Normal	461 (33.3%)	197 (33.3%)	
Moderate	462 (33.4%)	198 (33.4%)	
Severe	462 (33.4%)	197 (33.3%)	
GNRI	98.9 ± 18.3	98.9 ± 17.9	0.999
GNRI_group, n (%)			<0.001
Normal	489 (35.3%)	283 (47.8%)	
Low	207 (14.9%)	92 (15.5%)	
Moderate	293 (21.2%)	134 (22.6%)	

Characteristic	Internal cohort (N=1,385)	External cohort (N=592)	P-value
Severe	396 (28.6%)	83 (14.0%)	
Vital signs (mean ± SD)			
HR, beats/min	89.8 ± 21.5	89.3 ± 20.7	0.630
NBPS, mmHg	121 ± 26.0	122 ± 25.3	0.430
NBPD, mmHg	69.5 ± 19.8	70.5 ± 20.8	0.319
RR (SpO_2 in data), %*	96.8 ± 23.9	97.7 ± 36.1	0.559
SpO_2 (RR in data), /min*	20.7 ± 6.59	21.0 ± 6.83	0.358
Temperature, °F	98.2 ± 3.23	98.2 ± 2.95	0.998
Laboratory parameters (mean ± SD)			
Lymphocyte count, $\times 10^9$/L	1.23 ± 1.22	1.16 ± 0.91	0.203
HCT, %	32.8 ± 7.45	32.7 ± 7.69	0.788
Hb, g/L	105 ± 24.7	105 ± 25.6	1.000
PLT, K/µL	200 ± 107	196 ± 106	0.456
RDW, %	15.5 ± 2.46	15.4 ± 2.46	0.410
RBC, $\times 10^6$/µL	3.54 ± 0.85	3.53 ± 0.87	0.814
WBC, K/µL	14.4 ± 10.3	13.7 ± 9.78	0.162
Neutrophil count, $\times 10^9$/L	11.7 ± 7.70	11.1 ± 7.13	0.101
ALB, g/L	29.5 ± 6.01	29.5 ± 6.03	1.000
AG, mEq/L	15.2 ± 5.06	14.9 ± 4.88	0.221
Ca, mg/dL	8.33 ± 0.82	8.35 ± 0.79	0.609

Characteristic	Internal cohort (N=1,385)	External cohort (N=592)	P-value
Cl, mEq/L	103 ± 6.86	103 ± 6.63	1.000
Glu, mg/dL	166 ± 85.5	160 ± 73.8	0.134
K, mEq/L	4.35 ± 0.80	4.35 ± 0.80	1.000
Na, mEq/L	139 ± 5.90	138 ± 5.63	0.002
TCO_2, mEq/L	24.2 ± 6.28	24.3 ± 6.28	0.745
Lac, mmol/L	2.54 ± 2.24	2.49 ± 2.19	0.648
PCO_2, mmHg	44.6 ± 13.2	45.0 ± 13.5	0.544
pH	7.34 ± 0.11	7.34 ± 0.11	1.000
PO_2, mmHg	115 ± 102	115 ± 102	0.962
INR	1.54 ± 0.84	1.57 ± 0.85	0.463
PT, seconds	16.8 ± 9.31	17.1 ± 9.41	0.516
APTT, seconds	40.7 ± 28.5	41.5 ± 30.4	0.586
ALT, IU/L	140 ± 483	126 ± 384	0.524
AST, IU/L	213 ± 975	165 ± 464	0.196
TB, mg/dL	1.17 ± 2.20	1.11 ± 2.06	0.566
CRE, mg/dL	1.72 ± 1.59	1.63 ± 1.32	0.225
BUN, mg/dL	34.8 ± 26.5	34.6 ± 25.3	0.877
LDH, U/L	548 ± 1172	499 ± 1049	0.379
Phosphate	4.09 ± 1.63	4.10 ± 1.56	0.899
Monocyte count, $\times 10^9$/L	0.96 ± 1.54	0.94 ± 0.84	0.766

Characteristic	Internal cohort (N=1,385)	External cohort (N=592)	P-value
Treatments, n (%)			
Ventilation	1323 (95.5%)	565 (95.4%)	0.960
CRRT	229 (16.5%)	93 (15.7%)	0.650
Sa (Spironolactone)	1266 (91.4%)	537 (90.7%)	0.607
VP (Vasopressors)	1139 (82.2%)	490 (82.8%)	0.753
GC	565 (40.8%)	251 (42.4%)	0.510
AHT	1219 (88.0%)	524 (88.5%)	0.749
ABX	1320 (95.3%)	571 (96.5%)	0.228
Outcomes			
28-day ICU mortality, n (%)	402 (29.0%)	192 (32.4%)	0.134

Table S7: Sensitivity and specificity trade- offs of the Random Forest model under different strategies

Strategy	Dataset	Sensitivity	Specificity	AUC
Original RF (default)	Training	0.166	0.994	0.807
	Test	0.118	0.972	0.754
	External	0.093	0.988	0.734
Class- weighted RF	Training	0.314	0.942	0.801
	Test	0.235	0.928	0.746

	External	0.206	0.954	0.727
Threshold- optimized RF (cutoff ≈ 0.3)	Test	0.285	0.897	0.754
	External	0.251	0.920	0.734
